# Factors and outcomes for placental anomalies: An umbrella review of systematic reviews and meta-analyses

**DOI:** 10.7189/jogh.14.04013

**Published:** 2024-01-19

**Authors:** Dazhi Fan, Dongxin Lin, Jiaming Rao, Pengsheng Li, Gengdong Chen, Zixing Zhou, Li Sun, Li Liu, Yubo Ma, Xiaoling Guo, Zhengping Liu

**Affiliations:** 1 Foshan Fetal Medicine Research Institute, Foshan Women and Children Hospital, Foshan, Guangdong, China; 2 Department of Obstetrics, Foshan Women and Children Hospital, Foshan, Guangdong, China; 3 Department of Library, Foshan Women and Children Hospital, Foshan, Guangdong, China; 4 Department of Library, the First Affiliated Hospital, College of Medicine, Zhejiang University, Hangzhou, Zhejiang, China; 5 Department of Epidemiology and Biostatistics, School of Public Health, Anhui Medical University, Hefei, Anhui, China

## Abstract

**Background:**

Placental anomalies, including placenta previa (PP), placenta accreta spectrum disorders (PAS), and vase previa (VP), are associated with several adverse foetal-neonatal and maternal complications. However, there is still a lack of robust evidence on the pathogenesis and adverse outcomes of the diseases. Through this umbrella review, we aimed to systematically review existing meta-analyses exploring the factors and outcomes for pregnancy women with placental anomalies.

**Methods:**

We searched PubMed, Embase, Web of Science, and the Cochrane Library from inception to February 2023. We used AMSTAR 2 to assess the quality of the reviews and estimated the pooled risk and 95% confidence intervals (CIs) for each meta-analysis.

**Results:**

We included 34 meta-analyses and extracted 55 factors (27 for PP, 22 for PAS, and 6 for VP) and 16 outcomes (12 for PP, and 4 for VP) to assess their credibility. Seven factors (maternal cocaine use (for PP), uterine leiomyoma (for PP), prior abortion (spontaneous) (PP), threatened miscarriage (PP), maternal obesity (PP), maternal smoking (PAS), male foetus (PAS)) had high epidemiological evidence. Twelve factors and six outcomes had moderate epidemiological evidence. Twenty-two factors and eight outcomes showed significant association, but with weak credibility.

**Conclusions:**

We found varying levels of evidence for placental anomalies of different factors and outcomes in this umbrella review.

**Registration:**

PROSPERO: CRD42022300160.

Placental anomalies are associated with several adverse foetal-neonatal and maternal complications. They have three principal types: placenta previa (PP), placenta accreta spectrum disorders (PAS), and vase previa (VP) [1]. According to existing systematic reviews, the prevalence of placental anomalies has been increasing over the past two decades due to the growing incidence of assisted reproductive technologies and caesarean section [2–5]. The cause of placental anomalies is multifactorial, as are the related adverse pregnancy outcomes [6]. In particular, several factors and adverse outcomes, such as smoking, endometriosis, caesarean delivery, and perinatal haemorrhage, preterm delivery, foetal death, have been proposed as being relate to placental anomalies [7–9]. However, robust evidence to the pathogenesis and adverse outcomes of the diseases remain largely unknown.

Numerous meta-analysis and systematic reviews have explored the factors and pregnancy outcomes linked to placental anomalies [9–11]. However, most are incomplete and controversial, as they are limited by excess significance and publication bias. To consolidate the data from these meta-analyses, two umbrella reviews reported the risk factors for PP [7] and PAS [8], respectively. Although they identified some risk factors, they were still incomplete due to limited environmental risk factors. They also did not evaluate other common factors for PP (alcohol, obesity, threatened miscarriage, etc.) and non-environmental factors for PAS (prior abortion, prior uterine artery embolization, smoking, etc.), nor did they summarise the adverse pregnancy outcomes for either placental anomaly type.

Therefore, to evaluate the strength of epidemiological evidence of the reported associations of various factors and pregnancy outcomes with placental anomalies, including PP, PAS, and VP, we conducted an umbrella review of the evidence across existing systematic reviews and meta-analyses. We formed the following PICO question (except for the intervention, which our study did not have): ‘How does the strength of epidemiological evidence for factors and outcomes (O) for placental anomalies (P) compared to normal pregnant women (C)’?

## METHODS

We conducted an umbrella review using standardised methodology and reported our findings according to the PRISMA and MOOSE guidelines [12,13]. The protocol was registered in PROSPERO (CRD42022300160).

### Search strategy

We searched PubMed, Embase, Web of Science, and the Cochrane Library from inception to 8 February 2023 to identify systematic reviews and meta-analyses of studies that analysed the association between factors and outcomes and placental anomalies, including PP, PAS, and VP. We conceptualised the search strategy around the following keywords: ‘morbidly adherent placenta,’ ‘abnormal placentation,’ ‘placenta previa,’ ‘placenta accreta spectrum disorders,’ or ‘vasa previa,’ combined with ‘systematic review’ or ‘meta-analysis’ (File S1 in the **Online Supplementary Document**). We did not impose any language limitations when choosing the appropriate studies. We also manually searched the reference lists of relevant reviews and performed forward and backward citation chaining. Two researchers (DF and LL) independently screened and evaluated the full texts of potentially eligible articles. Disagreements were resolved through discussion.

### Inclusion and exclusion criteria

We included all meta-analyses of observational studies that investigated the causes and consequences of PP, PAS, and VP, irrespective of their publication date. We excluded systematic reviews without meta-analyses, animal studies, genetic studies, conference abstracts, letters, and editorials (File S2 in the **Online Supplementary Document**). If we found two similar articles, we included the most recent in the analysis, as it likely comprises more studies and/or is of the highest quality per the AMSTAR 2 tool [14].

### Data extraction

Two researchers (DF and JR) extracted the following data independently: first author, journal and year of publication, factor(s) and outcome(s) of interest (PP, PAS, or VP), and number of studies analysed. Where possible, they extracted other data such as estimated value, 95% confidence intervals (CIs), *P*-values, the number of participants in each groups, analytical data model, heterogeneity, and small-study effects. Disagreements in the extraction process were resolved through consensus.

### Quality of meta-analyses

Two investigators (DF and YM) assessed the methodological quality of each included meta-analysis using the AMSTAR 2 tool, a reliable and valid instrument facilitates the quality assessment of meta-analysis and systematic reviews [14–16] (File S3 in the **Online Supplementary Document**). AMSTAR 2 categorises the quality of a meta-analysis on a scale from critically low to high, based on 16 predefined items [17]. Each item has three responses – yes, partial yes, and no; items 2, 4, 7, 9, 11, 13, and 15 are key to the evaluation. Overall, the included articles are ranked as high, moderate, low, or critically low.

### Determining the credibility of evidence

We noted which associations met the following criteria to determine the strength of the epidemiologic evidence (i.e. the confidence in the effect estimate): Precision of the estimate (i.e. *P*-value <0.001, a threshold associated with significantly less false-positive results, and over 1000 cases with the disease); consistency of results (*I*^2^<50% and Cochran’s Q test *P*-value >0.10); and no evidence of small-study effects (*P*-value >0.10) [18–20].

We ranked the strength of epidemiologic evidence as high (when all the above criteria were satisfied), moderate (if a maximum of one criterion was not satisfied and a *P*-value <0.001), or weak (*P*-value <0.05). If the *P*-value was not reported, we calculated the 95% CI of the pooled effect estimate using a standard method.

### Statistical analysis

We displayed the derived random-effects estimates using forest plots to show the relationships between various factors or outcomes and PP, PAS, or VP. We computed and presented the random-effects estimate whenever a fixed-effects model was originally used. In practice, these different measures (rate ratio (RR), odds ratio (OR), and hazard ratio (HR)) of effect yield similar estimates, since PP, PAS, or VP is a rare occurrence. We converted these various measurements to ORs using a standardised methodology [21]. We used the Shapiro-Wilk test was used to test for normality of continuous variables and presented data with skewed distribution using medians and interquartile ranges (IQRs). We considered *P*-value <0.05 statistically significant, except for heterogeneity and small-study effects. All statistical analyses were conducted in Stata software, version 12.0 (StataCorp, College Station, Texas, USA).

## RESULTS

### Search results

**Figure 1:**
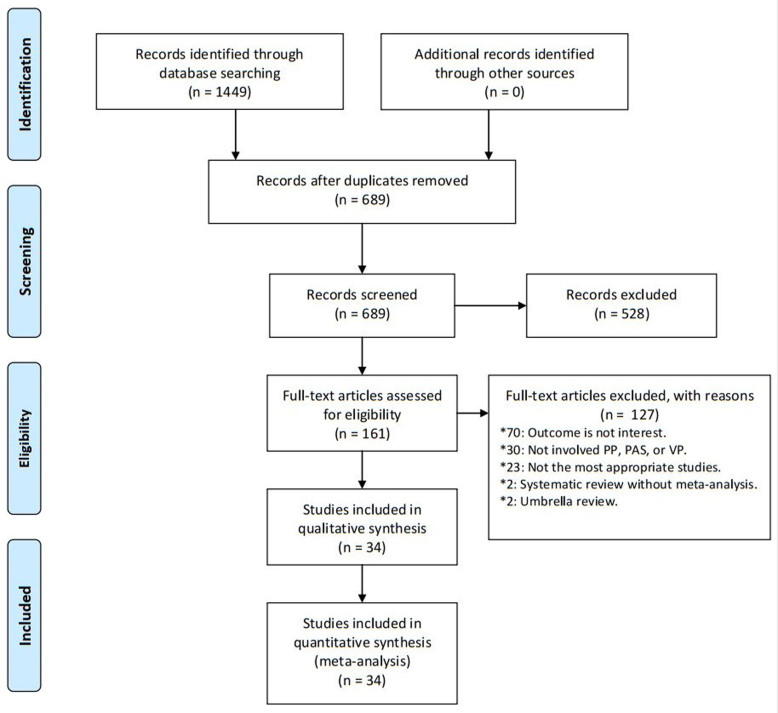
Flowchart of literature search.

We retrieved 689 studies, of which 34 met the eligibility criteria (**Figure 1**). They were published between 2003 and 2023. Twenty-seven articles focussed on PP [9,10,15–17,22–43], eight on PAS [10,11,25,44–48], and only two on VP [9,49]. Two articles [10,25] focussed on PP and PAS and one [9] on PP and VP. We found 55 factors (27 for PP, 22 for PAS, and 6 for VP) and 16 outcomes (12 for PP, and 4 for VP) in the 34 included studies. No meta-analysis evaluated the outcomes for PAS. There was a median of six primary studies per evidence synthesis (IQR = 3–12) with a median number of 1772 cases (IQR = 359–9532) and 278 459 subjects (IQR = 48 209–1 055 206) (**Table 1**).

**Table 1:** Characteristics, quantitative synthesis, and assessment of the included meta-analyses

					Random effects model		Heterogeneity				
**Factors/outcomes**	**Author (year)**	**Number of studies**	**Number of cases**	**Number of subjects**	**OR (95% CI)**	*P*-value	***I* ^2^**	***P*-value**	**Small study effects**	**AMSTAR 2**	**Credibility of the evidence**
**Factors for PP**											
AMA (>45 y)	Sugai et al. (2023) [41]	14	101 794	14 604 565	3.61 (2.70–4.81)	0.0001	81.4%	0.0001	0.459	High	Moderate
ART (programmed frozen ET)	Busnelli et al. (2022) [23]	10	642	75 973	1.12 (0.88–1.43)	0.342	37.3	0.0485	0.869	Low	NS
IMH	Zhuo et al. (2022) [42]	4	941	36 683	1.79 (0.64–5.01)	0.218	63.2	0.043	0.270	Low	NS
Maternal alcohol	Steane et al. (2021) [10]	4	16 071	6 984 743	1.27 (1.00–1.60)	0.047	62.5	0.046	0.482	High	Weak
Endometriosis	Matsuzaki et al. (2021) [24]	19	53 084	7 282 776	3.53 (3.00–4.16)	0.0001	72.6	0.0001	0.036	High	Weak
Adolescent pregnancy	Karacam Z, 2021 [26]	7	126	25 696	0.52 (0.31–0.88)	0.010	0	0.580	0.562	High	Weak
Prior UAE	Matsuzaki et al. (2021) [25]	4	2220	320 906	5.66 (1.78–18.02)	0.003	55	0.083	0.062	Low	Weak
Anti-TNF in IBD	Tandon et al. (2020) [27]	2	5	1188	1.58 (0.30–8.48)	0.591	0	0.554	-	Low	NS
Uterine leiomyoma	Jenabi et al. (2019) [28]	9	1772	255 886	2.29 (1.64–3.20)	0.0001	33.2	0.152	0.281	Low	High
ART (endometriosis)	Horton et al. (2019) [15]	6	164	4646	3.31 (1.26–8.71)	0.020	80	0.0001	0.335	High	Weak
AMA (>35 y)	Martinelli et al. (2018) [31]	23	113 990	21 961 192	3.16 (2.79–3.57)	0.0001	96.5	0.0001	0.318	High	Moderate
ART (singleton pregnancy)	Karami et al. (2018) [32]	14	3846	965 379	2.80 (1.99–3.61)	0.0001	72.7	0.0001	0.986	Low	Moderate
ART (twin pregnancy)	Karami et al. (2018) [32]	9	87	17 063	2.56 (0.97–4.14)	0.002	0	0.990	0.706	Low	Weak
ART (fresh ET)	Sha et al. (2018) [30]	6	816	72 584	1.63(1.13–2.36)	0.009	65	0.014	0.026	Low	Weak
Maternal smoking	Shobeiri et al. (2017) [33]	21	16 878	9 094 443	1.35 (1.27–1.44)	0.0001	62.8	0.0001	0.026	Low	Weak
Prior abortion (spontaneous)	Karami et al. (2017) [16]	16	3036	58 713	1.77 (1.60–1.94)	0.0001	0	0.652	0.742	Low	High
Prior abortion (induced)	Karami et al. (2017) [16]	10	2946	62 459	1.36 (1.02–1.69)	0.0001	59.2	0.009	0.486	Low	Moderate
HDP	Yin et al. (2015) [17]	7	2583	505 738	0.53 (0.30–0.94)	0.029	85.5	0.0001	0.590	Critically low	Weak
Maternal asthma	Wang et al. (2014) [34]	8	3793	1 359 749	1.19 (1.04–1.37)	0.010	0	0.599	0.412	Low	Weak
CHB infection	Huang et al. (2014) [35]	7	9119	1 687 276	1.77 (0.69–4.59)	0.237	53.5	0.044	0.087	Low	NS
eSET	Grady et al. (2012) [36]	1	72	15 306	6.02 (2.74–13.25)	0.0001	-	-	-	Low	Weak
Prior CS	Gurol-Urganci et al. (2011) [37]	37	9532	399 674	1.79 (1.60–1.98)	0.0001	82	0.0001	0.027	Low	Weak
Miscarriage (threatened)	Saraswat et al. (2010) [38]	6	229	64 365	1.62 (1.19–2.21)	0.0001	0	0.477	0.706	Low	High
Maternal obesity	Heslehurst et al. (2008) [39]	7	2647	756 217	0.83 (0.71–0.96)	0.0001	0	0.526	0.305	Low	High
Maternal cocaine use	Faiz et al. (2003) [40]	3	359	55 562	2.91 (1.90–4.29)	0.0001	0	0.526	0.448	Critically low	High
Male foetus	Faiz et al. (2003) [40]	7	3620	798 119	1.20 (1.12–1.31)	0.0001	41.9	0.215	0.0001	Critically low	Weak
Preeclampsia	Faiz et al. (2003) [40]	3	445	37 922	0.89 (0.51–1.41)	0.546	50.2	0.046	0.334	Critically low	NS
**Outcomes for PP**											
Blood transfusion (CS)	Iqbal et al. (2022) [22]	17	10 903	384 949	7.62 (5.79–10.03)	0.0001	88.8	0.0001	0.141	Low	Moderate
Preterm delivery (<37 weeks)	Jansen et al. (2022) [43]	12	2 247 569	23 664 492	9.51 (7.60–11.91)	0.0001	97.1	0.0001	0.028	High	Weak
Preterm delivery (<34 weeks)	Jansen et al. (2022) [43]	5	611 191	22 444 795	6.12 (4.29–8.72)	0.0001	90.5	0.0001	0.999	High	Moderate
Preterm delivery (<32 weeks)	Jansen et al. (2022) [43]	4	337 186	22 861 089	8.58 (6.35–11.58)	0.0001	88.7	0.0001	0.377	High	Moderate
Preterm delivery (<28 weeks)	Jansen et al. (2022) [43]	4	98 186	22 792 315	5.61 (4.02–7.83)	0.0001	61.6	0.050	0.193	High	Moderate
IUGR	Balayla et al. (2019) [29]	13	10 575	1 593 226	1.31 (0.98–1.75)	0.071	92	0.0001	0.498	Low	NS
NICU admission	Vahanian et al. (2015) [9]	5	48 915	844 906	4.09 (2.75–6.09)	0.0001	96.6	0.0001	0.777	Low	Moderate
Neonatal death	Vahanian et al. (2015) [9]	3	57 765	22 929 501	5.43 (3.03–9.74)	0.0001	88.7	0.0001	0.594	Low	Moderate
Perinatal death	Vahanian et al. (2015) [9]	3	5422	597 163	3.00 (1.38–6.54)	0.006	88.6	0.0001	0.553	Low	Weak
SGA	Vahanian et al. (2015) [9]	5	146 039	1 137 103	0.97 (0.67–1.41)	0.875	90.5	0.0001	0.526	Low	NS
APGAR-1 < 7	Vahanian et al. (2015) [9]	2	20 155	278 459	3.15 (1.69–5.88)	0.0001	93.9	0.0001	-	Low	Weak
APGAR-5 < 7	Vahanian et al. (2015) [9]	3	1839	635 703	2.73 (2.25–3.29)	0.039	98.7	0.0001	0.652	Low	Weak
**Factors for PAS**											
Maternal smoking	Jenabi et al. (2022) [45]	14	9800	3 892 832	1.21 (1.02–1.41)	0.0001	4.7	0.400	0.439	Low	High
HDP	Li et al. (2022) [44]	6	816	126 224	0.74 (0.38–1.44)	0.379	54.1	0.054	0.317	Low	NS
Prior UAE	Matsuzaki et al. (2021) [25]	3	55	3236	25.83 (10.87–61.37)	0.0001	0	0.677	0.062	Low	Moderate
ART	Matsuzaki et al. (2021) [46]	9	1081	206 634	5.03 (3.34–7.56)	0.0001	76.4	0.0001	0.008	Low	Weak
Maternal alcohol	Steane et al. (2021) [10]	1	350	79 393	0.92 (0.46–1.86)	0.814	-	-	-	High	NS
Male foetus	Hou et al. (2020) [47]	5	1856	804 043	0.79 (0.74–0.84)	0.0001	0	0.0001	0.953	High	High
Multiple gestations	Hou et al. (2020) [47]	7	147	30 458	1.79 (0.91–2.66)	0.0001	80.5	0.0001	0.310	High	Moderate
Low SES	Hou et al. (2020) [47]	3	390	244 792	0.47 (0.26–0.67)	0.0001	87.3	0.0001	0.162	High	Moderate
Maternal obesity	Iacovelli et al. (2020) [11]	5	516	554 106	1.33 (1.02–1.74)	0.038	0	0.543	0.893	High	Weak
AMA	Iacovelli et al. (2020) [11]	17	1152	1 055 206	2.40 (1.12–5.16)	0.024	96.1	0.0001	0.038	High	Weak
Prior uterine surgery	Iacovelli et al. (2020) [11]	34	1869	1 057 363	3.04 (2.16–4.29)	0.0001	77	0.0001	0.626	High	Moderate
Prior CS	Iacovelli et al. (2020) [11]	33	1662	656 168	3.12 (2.14–4.55)	0.0001	78.3	0.0001	0.956	High	Moderate
PP	Iacovelli et al. (2020) [11]	24	1694	1 057 222	4.75 (2.06–10.93)	0.0001	96.8	0.0001	0.145	High	Moderate
Multiparity	Iacovelli et al. (2020) [11]	19	1559	1 022 765	1.95 (1.43–2.65)	0.0001	70.9	0.0001	0.718	High	Moderate
PP and prior CS	Iacovelli et al. (2020) [11]	12	331	429 007	6.91 (1.29–37.08)	0.024	96.1	0.0001	0.037	High	Weak
Prior curettage	Iacovelli et al. (2020) [11]	16	644	10 886	1.54 (0.91–2.62)	0.109	78.9	0.0001	0.910	High	NS
Prior myomectomy	Iacovelli et al. (2020) [11]	9	309	938	0.76 (0.35–1.66)	0.486	0	0.617	0.557	High	NS
Prior abortion	Iacovelli et al. (2020) [11]	6	543	36 111	1.22 (0.87–1.71)	0.243	40.5	0.135	0.583	High	NS
Prior CS (elective)	Iacovelli et al. (2020) [11]	3	506	693 724	2.47 (0.17–36.67)	0.512	99.2	0.0001	0.290	High	NS
Prior CS (emergency)	Iacovelli et al. (2020) [11]	3	316	606 098	1.41 (0.33–6.03)	0.642	96.2	0.0001	0.101	High	NS
SISP	Iacovelli et al. (2020) [11]	2	143	820	1.60 (0.63–4.10)	0.324	78.3	0.032	-	High	NS
ART (frozen ET)	Roque et al. (2018) [48]	2	149	48 209	3.51 (2.04–6.05)	0.0001	0	0.553	-	Low	Moderate
**Factors for VP**											
STPP	Ruiter et al. (2016) [49]	4	1231	202 296	18.97 (6.13–58.68)	0.0001	66	0.030	0.282	High	Weak
VCI	Ruiter et al. (2016) [49]	2	161	20 634	93.57 (25.29–346.21)	0.0001	0	0.580	-	High	Weak
ART	Ruiter et al. (2016) [49]	2	1997	84 881	18.95 (6.61–54.34)	0.0001	29	0.240	-	High	Weak
Bilobed placenta	Ruiter et al. (2016) [49]	2	72	19 776	55.84 (11.89–262.26)	0.0001	0	0.999	-	High	Weak
CILTTU	Ruiter et al. (2016) [49]	2	61	5010	279.28 (1.51–51547.34)	0.030	86	0.007	-	High	Weak
Multiple gestations	Ruiter et al. (2016) [49]	3	627	16 660	3.14 (0.97–10.11)	0.055	0	0.454	-	High	NS
**Outcomes for VP**											
Preterm delivery	Vahanian et al. (2015) [9]	1	21 743	246525	3.36 (2.76–4.09)	0.0001	-	-	-	Low	Weak
SGA	Vahanian et al. (2016) [9]	1	5192	246 525	4.02 (2.64–6.12)	0.0001	-	-	-	Low	Weak
Perinatal death	Vahanian et al. (2015) [9]	1	3463	246 525	4.52 (2.77–7.39)	0.0001	-	-	-	Low	Weak
APGAR-5 < 7	Vahanian et al. (2015) [9]	1	7651	246 525	2.18 (1.36–3.50)	0.003	-	-	-	Low	Weak

AMA – advanced maternal age, APGAR – appearance, pulse, grimace, activity, and respiration, ART – assisted reproductive techniques, CHB – chronic hepatitis B, CS – caesarean section, eSET – elective single embryo transfer, ET – embryo transfer, HDP – hypertensive disorders of pregnancy, NS – not significant, OR – odds ratio, PAS – placenta accreta spectrum disorders, PP – placenta previa, SES – socioeconomic status, SGA – small for gestational age, SISP – short interval between prior caesarean section and subsequent pregnancy (<23 mo), STPP – second trimester placenta previa, TNF – tumour necrosis factor, UAE – uterine artery embolization, VCI – velamentous cord insertion, VP – vasa previa, wk – weeks.

### Quality assessment of meta-analyses

Eleven studies were of ‘high’ (32.35%), twenty-one of ‘low’ (61.77%), and two of ‘critically low’ quality (6.45%). The most frequent flaw was the absence of a registered protocol (item 2: 23 meta-analyses (67.65%)) and inadequacy of the literature search (item 4: 2 meta-analyses (5.88%)) (File S3 in the **Online Supplementary Document**).

### Strength of epidemiologic evidence

**Figure 2:**
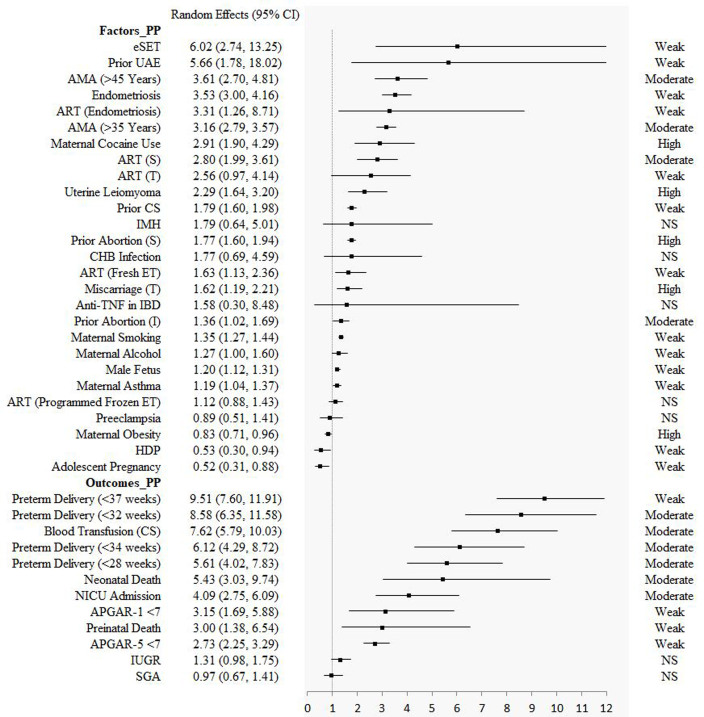
The results of factors and outcomes for placenta previa. AMA – advanced maternal age, ART – assisted reproductive techniques, CHB – chronic hepatitis B, CI – confidence interval, CS – caesarean section, eSET – elective single embryo transfer, ET – embryo transfer, HDP – hypertensive disorders of pregnancy, IBD – inflammatory bowel disease, IMH – isolated maternal hypothyroxinaemia, IUGR – intrauterine growth restriction, NICU – neonatal intensive care unit, NS – not significant, PP – placenta previa, SGA – small for gestational age, TNF – tumour necrosis factor, UAE – uterine artery embolization.

**Figure 3:**
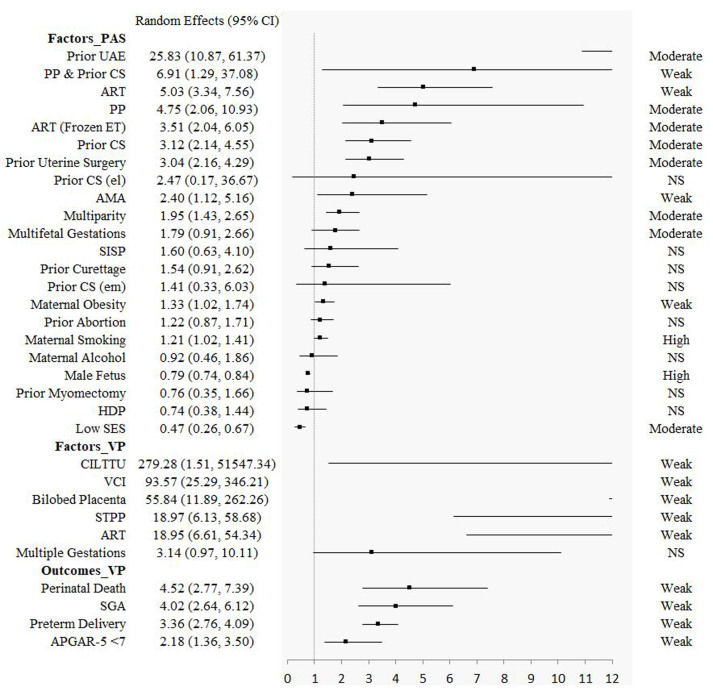
The results of factors for placenta accrete spectrum disorders and the factors and outcomes for vasa previa. AMA – advanced maternal age, ART – assisted reproductive techniques, CI – confidence interval, CILTTU – cord insertion in the lower third of the uterus at first trimester ultrasound, CS – caesarean section, ET – embryo transfer, HDP – hypertensive disorders of pregnancy, NS – not significant, PAS – placenta accreta spectrum disorders, PP – placenta previa, SES – socioeconomic status, SGA – small for gestational age, SISP – short interval between prior caesarean section and subsequent pregnancy (<23 months), STPP – second trimester placenta previa, UAE – uterine artery embolization, VCI – velamentous cord insertion, VP – vasa previa.

Seven factors (maternal cocaine use (for PP), uterine leiomyoma (for PP), prior abortion (spontaneous) (PP), threatened miscarriage (PP), maternal obesity (PP), maternal smoking (PAS), male foetus (PAS)) had high epidemiological evidence. Twelve factors (advanced maternal age (>45 years) (PP), advanced maternal age (>35 years) (PP), assisted reproductive techniques (singleton pregnancy) (PP), prior abortion (induced) (PP), prior uterine artery embolization (PAS), placenta previa (PAS), assisted reproductive techniques (frozen embryo transfer) (PAS), prior caesarean section (PAS), prior uterine surgery (PAS), multiparity (PAS), multiple gestations (PAS), low socioeconomic status (PAS)) and six outcomes (preterm delivery (<32 weeks) (PP), blood transfusion in caesarean section (PP), preterm delivery (<34 weeks) (PP), preterm delivery (<28 weeks) (PP), neonatal death (PP), neonatal intensive care unit (PP)) had moderate epidemiological evidence. Twenty-two factors (13 for PP, 4 for PAS, and 5 for VP) and eight outcomes (4 for PP, and 4 for VP) showed significant association, but with weak credibility. Other fourteen factors (5 for PP, 8 for PAS, and 1 for VP) and two outcomes (for PP) showed no statistically significant estimates (**Table 1**, **Figure 2**, and **Figure 3**; File S4 in the **Online Supplementary Document**).

## DISCUSSION

In this umbrella review, we identified seven factors demonstrating high strength of epidemiologic evidence, as well as twelve factors and six outcomes demonstrating moderate strength of epidemiologic evidence. Meanwhile, the estimate of effect’s degree of confidence was weaker another 22 factors and 8 outcomes. The methodological quality across meta-analyses differed slightly.

A previous umbrella study has attempted to explore the risk factors for PP [7] and found seven high and two weak risk factors. We evaluated a further sixteen factors and found two which had two high epidemiological evidence (threatened miscarriage and maternal obesity). We also twelve outcomes – six moderate, four weak, and two of insignificant epidemiologic evidence. Compared to previous study [7], we not only explored risk factors, but also reported outcomes for PP, addressing the existing gap in the literature.

A previously published umbrella review evaluated and found seven environmental risk factors for PAS [8]; in our study, we found another 15 – one of high, five of moderate, three of weak, and six of statistically insignificant epidemiological evidence.

The factors found in the previous umbrella review and our study differ slightly. This includes four factors for PP (hypertensive disorders of pregnancy; assisted reproductive techniques (singleton pregnancy); assisted reproductive techniques (twin pregnancy); and endometriosis) and one for PAS (hypertensive disorders of pregnancy). This is mainly caused by the different included articles. For example – regarding hypertension in pregnancy in PAS, we selected the latest study by Li et al. [44] rather than the one by Wang et al. [50] study. For endometriosis in PP, we chose the study by Matsuzaki et al. [24], not the one by Zullo et al. [51]. We determined by detailed evaluation and comparison that the two studies by Li et al. [44] and Matsuzaki et al. [24] are more recent and have higher quality (per the AMSTAR 2 tool), and are also more in line with our inclusion criteria.

VP is a rare but life-threatening obstetric disease, with an incidence from 0.46 to 0.60 for every 1000 deliveries, according to two systematic reviews [5,49]. Each effect size is relatively large for the six factors and four outcomes in VP. However, the epidemiologic evidence for this estimation was weak, mainly because the sample size of the included studies was too small. Therefore, more research is needed for this rare obstetrical condition. While three other systematic reviews and meta-analyses assessed VP [5,52,53], their topics were not related to and could not be evaluated in this umbrella review.

The previous review noted antepartum haemorrhage, postpartum haemorrhage, and septicaemia (among others) as adverse pregnancy outcomes for placental anomalies [8]. However, we did not find several of these outcomes in our review. Therefore, more systematic reviews and meta-analyses are needed to evaluate the effect of placental anomalies on other adverse pregnancy outcomes in the future.

The AMSTAR 2 tool assisted us in evaluating the methodological quality of the included studies. The most common flaw and reason for downgrading the quality assessment was the lack of protocol (n = 23). The most recent registration among the included studies took place in 2016. While preregistration of study protocols was an uncommon practice until recently, especially in the field of obstetrics, we found that implementing it could improve the quality of published meta-analysis.

To our knowledge, this is the first umbrella review to provide a broad overview of the scope and validity of the reported associations of various factors and pregnancy outcomes with placental anomalies, including PP, PAS, and VP. However, some limitations should be considered. First, our results exclusively rely on meta-analyses of observational studies, and are thus subject to the same limitations – including over/under-reporting, recall bias, and reverse causation. Second, because most of included meta-analysis were of low quality according to the AMSTAR 2 assessment, the results should be interpreted with caution. Third, we found an uneven covariate mix across primary studies, which could have affected our findings so that it might be difficult to assess both the effect size or its direction. Fourth, the results may not necessarily correspond well with the clinical studies. Finally, due to the lack of raw data, we are not able to conduct further analyses.

## CONCLUSIONS

In this review, we provide a comprehensive overview and critical evaluation of the contributing factors and outcomes of placental anomalies. Across 55 factors and 16 outcomes, seven (five for PP and two for PAS) and 12 factors (four for PP, and eight for PAS) and six outcomes (for PP) showed high/moderate epidemiologic evidence for placental anomalies. The results can be used to reassure women or refer them to to pre-conception counselling clinics or antenatal clinics. Clinicians should consider and communicate these factors and outcomes when counselling their patients. Regarding future research, more broadly implementing the reporting criteria and registering observational studies that test hypotheses could help strengthen the evidence. Likewise, new meta-analyses are needed to obtain, evaluate, and validate the novel and strongest evidence.

## Additional material


Online Supplementary Document
