# Supplementary material for: Factors and outcomes for placental anomalies: An umbrella review of systematic reviews and meta-analyses
Source: J Glob Health. 2024 Jan 19;14:04013. doi: 10.7189/jogh.14.04013 (PMC10795857; doi:10.7189/jogh.14.04013)
Supplement: Online Supplementary Document [file jogh-14-04013-s001.pdf]

Supplementary Files

Supplementary File 1. Search strategy used in the umbrella review. (P2)

Supplementary File 2. Systematic reviews and meta-analyses that excluded with reasons. (P3)

Supplementary file 3: AMSTAR-2 Results. (P15)

Supplementary file 4. The detailed information of the factors and outcomes for placental anomalies (PP, PAS, and VP) (P18)

Supplementary File 1. Search strategy used in the umbrella review.

1. “morbidly adherent placenta” OR “Placenta previa” OR “Placental previa” OR “Placenta praevia” OR “Placental disorders” OR “placentae previa” OR “low lying placenta”

2. “placenta accreta spectrum” OR “abnormally invasive placenta” OR “placenta accreta” OR “placenta increta” OR “placenta percreta” OR “morbidity adherent placenta” OR “morbidly adherent placenta” OR “invasive placenta” OR “inplatation placenta”

3. “vasa previa” OR “vasa praevia”

4. “Systematic review” OR Meta-analysis OR “Meta analysis” OR synthesis

5. (1 OR 2 OR 3) AND 4

Supplementary File 2. Systematic reviews and meta-analyses that excluded with reasons.

References	Reasons for exclusion
Takemoto Y, 2023[1]	Outcome is not interest.
Matsuzaki S, 2022[2]	Outcome is not interest.
Miller SE, 2023[3]	Outcome is not interest.
Kallianidis AF, 2023[4]	PP, PAS, or VP is not involved.
Siargkas A, 2022[5]	PP, PAS, or VP is not involved.
Siargkas A, 2022[6]	PP, PAS, or VP is not involved.
Rassie K, 2022[7]	PP, PAS, or VP is not involved.
Oltean I, 2022[8]	PP, PAS, or VP is not involved.
Nabhan AE, 2022[9]	Outcome is not interest.
Lin H, 2022[10]	Outcome is not interest.
Hessami K, 2022[11]	Outcome is not interest.
Fernandez-Jimenez N, 2022[12]	Outcome is not interest.
Alam B, 2022[13]	Outcome is not interest.
Spinillo SL, 2022[14]	Outcome is not interest.
Qu H, 2022[15]	Outcome is not interest.
Omar M, 2022[16]	PP, PAS, or VP is not involved.
Mitchell SJ, 2022[17]	Outcome is not interest.
Jenabi E, 2022[18]	It is an umbrella review.
Jenabi E, 2022[19]	It is an umbrella review.
Guzman Lopez JA, 2022[20]	Outcome is not interest.
Cavoretto PI, 2022[21]	Outcome is not interest.
Busnelli A, 2022[22]	It is not the most appropriate studies.
Alzoubi O, 2022[23]	Outcome is not interest.
Alves Junior JM, 2022[24]	Outcome is not interest.
Zhong W, 2021[25]	Outcome is not interest.
Zhang W, 2021[26]	Outcome is not interest.
Wang JQ, 2021[27]	It is not the most appropriate studies.
Vitale SG, 2021[28]	PP, PAS, or VP is not involved.
Tinari S, 2021[29]	Outcome is not interest.
Timor-Tritsch I, 2021[30]	It is not the most appropriate studies.
Soheili M, 2021[31]	PP, PAS, or VP is not involved.
Racher ML, 2022[32]	Outcome is not interest.
Novoa RH, 2021[33]	Outcome is not interest.
Neef V, 2021[34]	Outcome is not interest.
Nankali A, 2021[35]	Outcome is not interest.
Liu C, 2021[36]	Outcome is not interest.
Lim S, 2021[37]	Outcome is not interest.
Liang D, 2021[38]	Outcome is not interest.
Kubler JM, 2021[39]	Outcome is not interest.
Kotlyar AM, 2021[40]	Outcome is not interest.
Jafari M, 2021[41]	Outcome is not interest.

Hu KL, 2021[42]	It is not the most appropriate studies.
Hou W, 2021[43]	Outcome is not interest.
Favilli A, 2021[44]	PP, PAS, or VP is not involved.
Ende HB, 2021[45]	Outcome is not interest.
Dai M, 2021[46]	Outcome is not interest.
Cruz-Lemini M, 2022[47]	Outcome is not interest.
Breintoft K, 2021[48]	It is not the most appropriate studies.
Yang M, 2020[49]	It is not the most appropriate studies.
Turner JM, 2020[50]	Outcome is not interest.
Suarez S, 2020[51]	Outcome is not interest.
Pergialiotis V, 2020[52]	Outcome is not interest.
Pavalagantharajah S, 2020[53]	Outcome is not interest.
Patrick HS, 2020[54]	PP, PAS, or VP is not involved.
Ortiz-Esquinas I, 2020[55]	Outcome is not interest.
Morlando M, 2020[56]	Outcome is not interest.
Khazaei S, 2020[57]	PP, PAS, or VP is not involved.
Huang Y, 2020[58]	Outcome is not interest.
Cutts JC, 2020[59]	PP, PAS, or VP is not involved.
Chaemsaithong P, 2020[60]	PP, PAS, or VP is not involved.
Broere-Brown ZA, 2020[61]	It is not the most appropriate studies.
Khazaei S, 2019[62]	PP, PAS, or VP is not involved.
Jauniaux E, 2019[63]	Outcome is not interest.
Jauniaux E, 2019[64]	Outcome is not interest.
Jansen C, 2019[65]	Outcome is not interest.
Jansen C, 2019[66]	PP, PAS, or VP is not involved.
He Q, 2019[67]	Outcome is not interest.
Ferreira R, 2019[68]	PP, PAS, or VP is not involved.
De Mucio B, 2019[69]	It is not the most appropriate studies.
D'Antonio F, 2019[70]	Outcome is not interest.
Chen L, 2019[71]	Outcome is not interest.
Carroll L, 2019[72]	PP, PAS, or VP is not involved.
Aukes AM, 2019[73]	Outcome is not interest.
Adane AA, 2019[74]	PP, PAS, or VP is not involved.
Workalemahu T, 2018[75]	PP, PAS, or VP is not involved.
Wang W, 2018[76]	It is not the most appropriate studies.
Wan Z, 2018[77]	It is not meta-analysis.
Shahin Y, 2018[78]	Outcome is not interest.
Roberge S, 2018[79]	PP, PAS, or VP is not involved.
Pagani G, 2018[80]	Outcome is not interest.
Ordonez CA, 2018[81]	Outcome is not interest.
Ordonez CA, 2018[82]	Outcome is not interest.
Lalani S, 2018[83]	It is not the most appropriate studies.
Kohn JR, 2018[84]	It is a systematic review without meta-analysis.

Keag OE, 2018[85]	It is not the most appropriate studies.
Glazier JD, 2018[86]	Outcome is not interest.
Glavind MT, 2018[87]	It is a systematic review without meta-analysis.
Gasparri ML, 2018[88]	It is not the most appropriate studies.
Familiari A, 2018[89]	Outcome is not interest.
D'Antonio F, 2018[90]	Outcome is not interest.
Cali G, 2018[91]	It is not the most appropriate studies.
Buca D, 2018[92]	Outcome is not interest.
Zullo F, 2017[93]	It is not the most appropriate studies.
Shobeiri F, 2017[94]	PP, PAS, or VP is not involved.
Jenabi E, 2017[95]	Outcome is not interest.
Jauniaux E, 2017[96]	Outcome is not interest.
Ismail KI, 2017[97]	PP, PAS, or VP is not involved.
Fan D, 2017[98]	Outcome is not interest.
Fan D, 2017[99]	Outcome is not interest.
Fan D, 2017[100]	Outcome is not interest.
van den Akker T, 2016[101]	PP, PAS, or VP is not involved.
Rodger MA, 2016[102]	Outcome is not interest.
Qin JB, 2016[103]	Outcome is not interest.
Qin J, 2016[104]	It is not the most appropriate studies.
Leone Roberti Maggiore U, 2016[105]	Outcome is not interest.
Fan D, 2016[106]	Outcome is not interest.
Di Mascio D, 2016[107]	Outcome is not interest.
Qin J, 2015[108]	It is not the most appropriate studies.
Marchi J, 2015[109]	It is not the most appropriate studies.
Duffy JMN, 2015[110]	PP, PAS, or VP is not involved.
Chibueze EC, 2015[111]	Outcome is not interest.
Tersigni C, 2014[112]	PP, PAS, or VP is not involved.
Klar M, 2014[113]	It is not the most appropriate studies.
Greenwood DC, 2014[114]	It is not the most appropriate studies.
Meng X, 2013[115]	Outcome is not interest.
Maheshwari A, 2013[116]	It is not the most appropriate studies.
Cresswell JA, 2013[117]	Outcome is not interest.
Rao KP, 2012[118]	Outcome is not interest.
Fekete K, 2012[119]	Outcome is not interest.
Abou-Nassar K, 2011[120]	PP, PAS, or VP is not involved.
Rodger MA, 2010[121]	PP, PAS, or VP is not involved.
Zdoukopoulos N, 2008[122]	PP, PAS, or VP is not involved.
Conde-Agudelo A, 2007[123]	It is not the most appropriate studies.
Castles A, 1999[124]	It is not the most appropriate studies.
Hulse GK, 1997[125]	PP, PAS, or VP is not involved.
Ananth CV, 1997[126]	It is not the most appropriate studies.
Ananth CV, 1996[127]	PP, PAS, or VP is not involved.

References

1. TAKEMOTO Y, MATSUZAKI S, MATSUZAKI S, et al. Current Evidence on Vasa Previa without Velamentous Cord Insertion or Placental Morphological Anomalies (Type III Vasa Previa): Systematic Review and Meta-Analysis. Biomedicines 2023;11.
2. MATSUZAKI S, UEDA Y, MATSUZAKI S, et al. The Characteristics and Obstetric Outcomes of Type II Vasa Previa: Systematic Review and Meta-Analysis. Biomedicines 2022;10.
3. MILLER SE, LEONARD SA, MEZA PK, et al. Red Blood Cell Transfusion in Patients With Placenta Accreta Spectrum: A Systematic Review and Meta-analysis. Obstetrics and gynecology 2023;141:49-58.
4. KALLIANIDIS AF, RIJNTJES D, BROBBEL C, DEKKERS OM, BLOEMENKAMP KWM, VAN DEN AKKER T. Incidence, Indications, Risk Factors, and Outcomes of Emergency Peripartum Hysterectomy Worldwide: A Systematic Review and Meta-analysis. Obstetrics and gynecology 2023;141:35-48.
5. SIARGKAS A, TSAKIRIDIS I, PACHI C, MAMOPOULOS A, ATHANASIADIS A, DAGKLIS T. Impact of velamentous cord insertion on perinatal outcomes: a systematic review and meta-analysis. American journal of obstetrics & gynecology MFM 2022;5:100812.
6. SIARGKAS A, TSAKIRIDIS I, GRAMMENOS P, et al. The impact of lateral placenta on preeclampsia and small for gestational age neonates: a systematic review and meta-analysis. Journal of perinatal medicine 2022.
7. RASSIE K, GIRI R, JOHAM AE, TEEDE H, MOUSA A. Human Placental Lactogen in Relation to Maternal Metabolic Health and Fetal Outcomes: A Systematic Review and Meta-Analysis. International journal of molecular sciences 2022;23.
8. OLTEAN I, RAJARAM A, TANG K, et al. The Association of Placental Abruption and Pediatric Neurological Outcome: A Systematic Review and Meta-Analysis. Journal of clinical medicine 2022;12.
9. NABHAN AE, ABDELQADIR YH, ABDELGHAFAR YA, et al. Therapeutic effect of Internal iliac artery ligation and uterine artery ligation techniques for bleeding control in placenta accreta spectrum patients: A meta-analysis of 795 patients. Frontiers in surgery 2022;9:983297.
10. LIN H, LI L, LIN Y, WANG W. Accuracy of Magnetic Resonance Imaging in Diagnosing Placenta Accreta: A Systematic Review and Meta-Analysis. Computational and mathematical methods in medicine 2022;2022:2751559.
11. HESSAMI K, SALMANIAN B, EINERSON BD, et al. Clinical Correlates of Placenta Accreta Spectrum Disorder Depending on the Presence or Absence of Placenta Previa: A Systematic Review and Meta-analysis. Obstetrics and gynecology 2022;140:599-606.
12. FERNANDEZ-JIMENEZ N, FORE R, CILLEROS-PORTET A, et al. A meta-analysis of pre-pregnancy maternal body mass index and placental DNA methylation identifies 27 CpG sites with implications for mother-child health. Communications biology 2022;5:1313.
13. ALAM B, NASIR F, AKBARI AR, ALALI B, KHALIL Z. A Review and Comparison of the Efficacy of Prophylactic Interventional Radiological Arterial Occlusions in Placenta Accreta Spectrum Patients: A Meta-analysis. Academic radiology 2022.
14. SPINILLO SL, FARINA A, SOTIRIADIS A, et al. Pregnancy outcome of confined placental mosaicism: meta-analysis of cohort studies. American journal of obstetrics and gynecology 2022.

15. Qu H, Du Y, Yu Y, Wang M, Han T, Yan L. The effect of endometriosis on IVF/ICSI and perinatal outcome: A systematic review and meta-analysis. Journal of gynecology obstetrics and human reproduction 2022;51:102446.
16. Omar M, Youssef MR, Trinh LN, et al. Excess of cesarean births in pregnant women with COVID-19: A meta-analysis. Birth 2022;49:179-93.
17. Mitchell SJ, Ngo G, Maurel KA, et al. Timing of birth and adverse pregnancy outcomes in cases of prenatally diagnosed vasa previa: a systematic review and meta-analysis. American journal of obstetrics and gynecology 2022;227:173-81 e24.
18. Jenabi E, Salimi Z, Salehi AM, Khazaei S. The environmental risk factors prior to conception associated with placenta accreta spectrum: An umbrella review. Journal of gynecology obstetrics and human reproduction 2022;51:102406.
19. Jenabi E, Salimi Z, Bashirian S, Khazaei S, Ayubi E. The risk factors associated with placenta previa: An umbrella review. Placenta 2022;117:21-27.
20. Guzman Lopez JA, Gutierrez Sanchez LA, Pinilla-Monsalve GD, Timor-Tritsch IE. Placenta accreta spectrum disorders in the first trimester: a systematic review. Journal of obstetrics and gynaecology : the journal of the Institute of Obstetrics and Gynaecology 2022:1-8.
21. Cavoretto PI, Giorgione V, Sotiriadis A, et al. IVF/ICSI treatment and the risk of iatrogenic preterm birth in singleton pregnancies: systematic review and meta-analysis of cohort studies. The journal of maternal-fetal & neonatal medicine : the official journal of the European Association of Perinatal Medicine, the Federation of Asia and Oceania Perinatal Societies, the International Society of Perinatal Obstet 2022;35:1987-96.
22. Busnelli A, Schirripa I, Fedele F, Bulfoni A, Levi-Setti PE. Obstetric and perinatal outcomes following programmed compared to natural frozen-thawed embryo transfer cycles: a systematic review and meta-analysis. Human reproduction 2022;37:1619-41.
23. Alzoubi O, Maaita W, Madain Z, et al. Association between placenta accreta spectrum and third-trimester serum levels of vascular endothelial growth factor, placental growth factor, and soluble Fms-like tyrosine kinase-1: A meta-analysis. The journal of obstetrics and gynaecology research 2022;48:2363-76.
24. Alves Junior JM, Bernardo WM, Ward LS, Villagelin D. Effect of Hyperthyroidism Control During Pregnancy on Maternal and Fetal Outcome: A Systematic Review and Meta-Analysis. Frontiers in endocrinology 2022;13:800257.
25. Zhong W, Zhu F, Li S, et al. Maternal and Neonatal Outcomes After Planned or Emergency Delivery for Placenta Accreta Spectrum: A Systematic Review and Meta-Analysis. Frontiers in medicine 2021;8:731412.
26. Zhang W, Geris S, Al-Emara N, Ramadan G, Sotiriadis A, Akolekar R. Perinatal outcome of pregnancies with prenatal diagnosis of vasa previa: systematic review and meta-analysis. Ultrasound in obstetrics & gynecology : the official journal of the International Society of Ultrasound in Obstetrics and Gynecology 2021;57:710-19.
27. Wang JQ, Zhang JM, Qian B. Adverse pregnancy outcomes for women with endometriosis: a systematic review and meta-analysis. Ginekologia polska 2021.
28. Vitale SG, Parry JP, Carugno J, et al. Surgical and Reproductive Outcomes after Hysteroscopic Removal of Retained Products of Conception: A Systematic Review and Meta-analysis. Journal of minimally invasive gynecology 2021;28:204-17.

29. TINARI S, BUCA D, CALI G, et al. Risk factors, histopathology and diagnostic accuracy in posterior placenta accreta spectrum disorders: systematic review and meta-analysis. Ultrasound in obstetrics & gynecology : the official journal of the International Society of Ultrasound in Obstetrics and Gynecology 2021;57:903-09.
30. TIMOR-TRITSCH I, BUCA D, DI MASCIO D, et al. Outcome of cesarean scar pregnancy according to gestational age at diagnosis: A systematic review and meta-analysis. European journal of obstetrics, gynecology, and reproductive biology 2021;258:53-59.
31. SOHEILI M, MORADI G, BARADARAN HR, SOHEILI M, MOKHTARI MM, MORADI Y. Clinical manifestation and maternal complications and neonatal outcomes in pregnant women with COVID-19: a comprehensive evidence synthesis and meta-analysis. The journal of maternal-fetal & neonatal medicine : the official journal of the European Association of Perinatal Medicine, the Federation of Asia and Oceania Perinatal Societies, the International Society of Perinatal Obstet 2021:1-14.
32. RACHER ML, MORRIS M, SCOTT AP, et al. Placental location site and adverse antepartum pregnancy complications: a meta-analysis and review of the literature. Archives of gynecology and obstetrics 2022;305:1265-77.
33. NOVOA RH, QUINTANA W, LLANCARI P, URBINA-QUISPE K, GUEVARA-RIOS E, VENTURA W. Maternal clinical characteristics and perinatal outcomes among pregnant women with coronavirus disease 2019. A systematic review. Travel medicine and infectious disease 2021;39:101919.
34. NEEF V, BUXMANN H, RABENAU HF, ZACHAROWSKI K, RAIMANN FJ. Characterization of neonates born to mothers with SARS-CoV-2 infection: Review and meta-analysis. Pediatrics and neonatology 2021;62:11-20.
35. NANKALI A, SALARI N, KAZEMINIA M, MOHAMMADI M, RASOULINYA S, HOSSEINIAN-FAR M. The effect prophylactic internal iliac artery balloon occlusion in patients with placenta previa or placental accreta spectrum: a systematic review and meta-analysis. Reproductive biology and endocrinology : RB&E 2021;19:40.
36. LIU C, YANG DD, QU HB, GUO Y, LIU LJ. Efficacy and safety of prophylactic abdominal aortic balloon occlusion versus internal iliac arterial balloon occlusion for placenta accreta spectrum disorder: A systematic review and meta-analysis. Clinical imaging 2021;78:250-55.
37. LIM S, LI W, KEMPER J, NGUYEN A, MOL BW, REDDY M. Biomarkers and the Prediction of Adverse Outcomes in Preeclampsia: A Systematic Review and Meta-analysis. Obstetrics and gynecology 2021;137:72-81.
38. LIANG D, ZHAO H, LIU D, LIN Y. Internal iliac artery balloon occlusion in the management of placenta accreta: A systematic review and meta-analysis. European journal of radiology 2021;139:109711.
39. KUBLER JM, CLIFTON VL, MOHOLDT T, BEETHAM KS. The effects of exercise during pregnancy on placental composition: A systematic review and meta-analysis. Placenta 2021;117:39-46.
40. KOTLYAR AM, GRECHUKHINA O, CHEN A, et al. Vertical transmission of coronavirus disease 2019: a systematic review and meta-analysis. American journal of obstetrics and gynecology 2021;224:35-53 e3.
41. JAFARI M, PORMOHAMMAD A, SHEIKH NESHIN SA, et al. Clinical characteristics and outcomes of pregnant women with COVID-19 and comparison with control patients: A

systematic review and meta-analysis. Reviews in medical virology 2021;31:1-16.
42. Hu KL, Zhang D, Li R. Endometrium preparation and perinatal outcomes in women undergoing single-blastocyst transfer in frozen cycles. Fertility and sterility 2021;115:1487-94.
43. Hou W, Shi G, Ma Y, et al. Impact of preimplantation genetic testing on obstetric and neonatal outcomes: a systematic review and meta-analysis. Fertility and sterility 2021;116:990-1000.
44. Favilli A, Tosto V, Ceccobelli M, et al. Risk factors for non-adherent retained placenta after vaginal delivery: a systematic review. BMC pregnancy and childbirth 2021;21:268.
45. Ende HB, Lozada MJ, Chestnut DH, et al. Risk Factors for Atonic Postpartum Hemorrhage: A Systematic Review and Meta-analysis. Obstetrics and gynecology 2021;137:305-23.
46. Dai M, Zhang F, Li K, Jin G, Chen Y, Zhang X. The effect of prophylactic balloon occlusion in patients with placenta accreta spectrum: a Bayesian network meta-analysis. European radiology 2022;32:3297-308.
47. Cruz-Lemini M, Vazquez JC, Ullmo J, Llurba E. Low-molecular-weight heparin for prevention of preeclampsia and other placenta-mediated complications: a systematic review and meta-analysis. American journal of obstetrics and gynecology 2022;226:S1126-S44 e17.
48. Breintoft K, Pinnerup R, Henriksen TB, et al. Endometriosis and Risk of Adverse Pregnancy Outcome: A Systematic Review and Meta-Analysis. Journal of clinical medicine 2021;10.
49. Yang M, Lin L, Sha C, et al. Which is better for mothers and babies: fresh or frozen-thawed blastocyst transfer? BMC pregnancy and childbirth 2020;20:559.
50. Turner JM, Robertson NT, Hartel G, Kumar S. Impact of low-dose aspirin on adverse perinatal outcome: meta-analysis and meta-regression. Ultrasound in obstetrics & gynecology : the official journal of the International Society of Ultrasound in Obstetrics and Gynecology 2020;55:157-69.
51. Suarez S, Conde-Agudelo A, Borovac-Pinheiro A, et al. Uterine balloon tamponade for the treatment of postpartum hemorrhage: a systematic review and meta-analysis. American journal of obstetrics and gynecology 2020;222:293 e1-93 e52.
52. Pergialiotis V, Bellos I, Fanaki M, Antsaklis A, Loutradis D, Daskalakis G. The impact of residual oligohydramnios following preterm premature rupture of membranes on adverse pregnancy outcomes: a meta-analysis. American journal of obstetrics and gynecology 2020;222:628-30.
53. Pavalagantharajah S, Villani LA, D'Souza R. Vasa previa and associated risk factors: a systematic review and meta-analysis. American journal of obstetrics & gynecology MFM 2020;2:100117.
54. Patrick HS, Mitra A, Rosen T, Ananth CV, Schuster M. Pharmacologic intervention for the management of retained placenta: a systematic review and meta-analysis of randomized trials. American journal of obstetrics and gynecology 2020;223:447 e1-47 e19.
55. Ortiz-Esquinas I, Rodriguez-Almagro J, Gomez-Salgado J, Arias-Arias A, Ballesta-Castillejos A, Hernandez-Martinez A. Effects of cord milking in late preterm infants and full-term infants: A systematic review and meta-analysis. Birth 2020;47:259-69.
56. Morlando M, Buca D, Timor-Tritsch I, et al. Reproductive outcome after cesarean scar pregnancy: A systematic review and meta-analysis. Acta obstetricia et gynecologica

Scandinavica 2020;99:1278-89.

57. KHAZAEI S, JENABI E. The association between polyhydramnios and the risk of placenta abruption: a meta-analysis. The journal of maternal-fetal & neonatal medicine : the official journal of the European Association of Perinatal Medicine, the Federation of Asia and Oceania Perinatal Societies, the International Society of Perinatal Obstet 2020;33:3035-40.
58. HUANG Y, ZHAO X, CHEN Y, WANG J, ZHENG W, CAO L. Miscarriage on Endometriosis and Adenomyosis in Women by Assisted Reproductive Technology or with Spontaneous Conception: A Systematic Review and Meta-Analysis. BioMed research international 2020;2020:4381346.
59. CUTTS JC, AGIUS PA, ZAW L, et al. Pregnancy-specific malarial immunity and risk of malaria in pregnancy and adverse birth outcomes: a systematic review. BMC medicine 2020;18:14.
60. CHAEMSAITHONG P, CUENCA-GOMEZ D, PLANA MN, GIL MM, POON LC. Does low-dose aspirin initiated before 11 weeks' gestation reduce the rate of preeclampsia? American journal of obstetrics and gynecology 2020;222:437-50.
61. BROERE-BROWN ZA, ADANK MC, BENSCHOP L, et al. Fetal sex and maternal pregnancy outcomes: a systematic review and meta-analysis. Biology of sex differences 2020;11:26.
62. KHAZAEI S, JENABI E, VEISANI Y. The association of Mullerian anomalies and placenta abruption: a meta-analysis. The journal of maternal-fetal & neonatal medicine : the official journal of the European Association of Perinatal Medicine, the Federation of Asia and Oceania Perinatal Societies, the International Society of Perinatal Obstet 2019;32:512-16.
63. JAUNIAUX E, GRONBECK L, BUNCE C, LANGHOFF-ROOS J, COLLINS SL. Epidemiology of placenta previa accreta: a systematic review and meta-analysis. BMJ open 2019;9:e031193.
64. JAUNIAUX E, BUNCE C, GRONBECK L, LANGHOFF-ROOS J. Prevalence and main outcomes of placenta accreta spectrum: a systematic review and meta-analysis. American journal of obstetrics and gynecology 2019;221:208-18.
65. JANSEN C, KLEINROUWELER CE, VAN LEEUWEN L, et al. Final outcome of a second trimester low-positioned placenta: A systematic review and meta-analysis. European journal of obstetrics, gynecology, and reproductive biology 2019;240:197-204.
66. JANSEN C, DE MOOIJ YM, BLOMAARD CM, et al. Vaginal delivery in women with a low-lying placenta: a systematic review and meta-analysis. BJOG : an international journal of obstetrics and gynaecology 2019;126:1118-26.
67. HE Q, LI YL, ZHU MJ, et al. Prophylactic abdominal aortic balloon occlusion in patients with pernicious placenta previa during cesarean section: a systematic review and meta-analysis from randomized controlled trials. Archives of gynecology and obstetrics 2019;300:1131-45.
68. FERREIRA R, NEGRINI R, BERNARDO WM, SIMOES R, PIATO S. The effects of sildenafil in maternal and fetal outcomes in pregnancy: A systematic review and meta-analysis. PloS one 2019;14:e0219732.
69. DE MUCIO B, SERRUYA S, ALEMAN A, CASTELLANO G, SOSA CG. A systematic review and meta-analysis of cesarean delivery and other uterine surgery as risk factors for placenta accreta. International journal of gynaecology and obstetrics: the official organ of the International Federation of Gynaecology and Obstetrics 2019;147:281-91.
70. D'ANTONIO F, IACOVELLI A, LIBERATI M, et al. Role of interventional radiology in pregnancy complicated by placenta accreta spectrum disorder: systematic review and meta-analysis. Ultrasound in obstetrics & gynecology : the official journal of the International Society of

Ultrasound in Obstetrics and Gynecology 2019;53:743-51.
71. Chen L, Wang X, Wang H, Li Q, Shan N, Qi H. Clinical evaluation of prophylactic abdominal aortic balloon occlusion in patients with placenta accreta: a systematic review and meta-analysis. BMC pregnancy and childbirth 2019;19:30.
72. Carroll L, Gallagher L, Smith V. Risk factors for reduced fetal movements in pregnancy: A systematic review and meta-analysis. European journal of obstetrics, gynecology, and reproductive biology 2019;243:72-82.
73. Aukes AM, Yurtsever FN, Boutin A, Visser MC, de Groot CJM. Associations Between Migraine and Adverse Pregnancy Outcomes: Systematic Review and Meta-analysis. Obstetrical & gynecological survey 2019;74:738-48.
74. Adane AA, Shepherd CCJ, Lim FJ, White SW, Farrant BM, Bailey HD. The impact of pre-pregnancy body mass index and gestational weight gain on placental abruption risk: a systematic review and meta-analysis. Archives of gynecology and obstetrics 2019;300:1201-10.
75. Workalemahu T, Enquobahrie DA, Gelaye B, et al. Genetic variations and risk of placental abruption: A genome-wide association study and meta-analysis of genome-wide association studies. Placenta 2018;66:8-16.
76. Wang W, Fan D, Wang J, et al. Association between hypertensive disorders complicating pregnancy and risk of placenta accreta: a meta-analysis and systematic review. Hypertension in pregnancy 2018;37:168-74.
77. Wan Z, Zhou A, Zhu H, et al. Maternal Hepatitis B Virus Infection and Pregnancy Outcomes: A Hospital-based Case-control Study in Wuhan, China. Journal of clinical gastroenterology 2018;52:73-78.
78. Shahin Y, Pang CL. Endovascular interventional modalities for haemorrhage control in abnormal placental implantation deliveries: a systematic review and meta-analysis. European radiology 2018;28:2713-26.
79. Roberge S, Bujold E, Nicolaides KH. Meta-analysis on the effect of aspirin use for prevention of preeclampsia on placental abruption and antepartum hemorrhage. American journal of obstetrics and gynecology 2018;218:483-89.
80. Pagani G, Cali G, Acharya G, et al. Diagnostic accuracy of ultrasound in detecting the severity of abnormally invasive placentation: a systematic review and meta-analysis. Acta obstetricia et gynecologica Scandinavica 2018;97:25-37.
81. Ordonez CA, Manzano-Nunez R, Parra MW, et al. Prophylactic use of resuscitative endovascular balloon occlusion of the aorta in women with abnormal placentation: A systematic review, meta-analysis, and case series. The journal of trauma and acute care surgery 2018;84:809-18.
82. Morfaw F, Fundoh M, Bartoszko J, Mbuagbaw L, Thabane L. Using tocolysis in pregnant women with symptomatic placenta praevia does not significantly improve prenatal, perinatal, neonatal and maternal outcomes: a systematic review and meta-analysis. Systematic reviews 2018;7:249.
83. Lalani S, Choudhry AJ, Firth B, et al. Endometriosis and adverse maternal, fetal and neonatal outcomes, a systematic review and meta-analysis. Human reproduction 2018;33:1854-65.
84. Kohn JR, Shamshirsaz AA, Popek E, Guan X, Belfort MA, Fox KA. Pregnancy after

endometrial ablation: a systematic review. BJOG : an international journal of obstetrics and gynaecology 2018;125:43-53.

85. Keag OE, Norman JE, Stock SJ. Long-term risks and benefits associated with cesarean delivery for mother, baby, and subsequent pregnancies: Systematic review and meta-analysis. PLoS medicine 2018;15:e1002494.

86. Glazier JD, Hayes DJL, Hussain S, et al. The effect of Ramadan fasting during pregnancy on perinatal outcomes: a systematic review and meta-analysis. BMC pregnancy and childbirth 2018;18:421.

87. Glavind MT, Mollgaard MV, Iversen ML, Arendt LH, Forman A. Obstetrical outcome in women with endometriosis including spontaneous hemoperitoneum and bowel perforation: a systematic review. Best practice & research Clinical obstetrics & gynaecology 2018;51:41-52.

88. Gasparri ML, Nirgianakis K, Taghavi K, Papadia A, Mueller MD. Placenta previa and placental abruption after assisted reproductive technology in patients with endometriosis: a systematic review and meta-analysis. Archives of gynecology and obstetrics 2018;298:27-34.

89. Familiari A, Liberati M, Lim P, et al. Diagnostic accuracy of magnetic resonance imaging in detecting the severity of abnormal invasive placenta: a systematic review and meta-analysis. Acta obstetricia et gynecologica Scandinavica 2018;97:507-20.

90. D'Antonio F, Timor-Tritsch IE, Palacios-Jaraquemada J, et al. First-trimester detection of abnormally invasive placenta in high-risk women: systematic review and meta-analysis. Ultrasound in obstetrics & gynecology : the official journal of the International Society of Ultrasound in Obstetrics and Gynecology 2018;51:176-83.

91. Cali G, Timor-Tritsch IE, Palacios-Jaraquemada J, et al. Outcome of Cesarean scar pregnancy managed expectantly: systematic review and meta-analysis. Ultrasound in obstetrics & gynecology : the official journal of the International Society of Ultrasound in Obstetrics and Gynecology 2018;51:169-75.

92. Buca D, Liberati M, Cali G, et al. Influence of prenatal diagnosis of abnormally invasive placenta on maternal outcome: systematic review and meta-analysis. Ultrasound in obstetrics & gynecology : the official journal of the International Society of Ultrasound in Obstetrics and Gynecology 2018;52:304-09.

93. Zullo F, Spagnolo E, Saccone G, et al. Endometriosis and obstetrics complications: a systematic review and meta-analysis. Fertility and sterility 2017;108:667-72 e5.

94. Shobeiri F, Masoumi SZ, Jenabi E. The association between maternal smoking and placenta abruption: a meta-analysis. The journal of maternal-fetal & neonatal medicine : the official journal of the European Association of Perinatal Medicine, the Federation of Asia and Oceania Perinatal Societies, the International Society of Perinatal Obstet 2017;30:1963-67.

95. Jenabi E, Ebrahimzadeh Zagami S. The association between uterine leiomyoma and placenta abruption: A meta-analysis. The journal of maternal-fetal & neonatal medicine : the official journal of the European Association of Perinatal Medicine, the Federation of Asia and Oceania Perinatal Societies, the International Society of Perinatal Obstet 2017;30:2742-46.

96. Jauniaux E, Bhide A. Prenatal ultrasound diagnosis and outcome of placenta previa accreta after cesarean delivery: a systematic review and meta-analysis. American journal of obstetrics and gynecology 2017;217:27-36.

97. Ismail KI, Hannigan A, O'Donoghue K, Cotter A. Abnormal placental cord insertion and

adverse pregnancy outcomes: a systematic review and meta-analysis. Systematic reviews 2017;6:242.

98. Fan D, Xia Q, Liu L, et al. The Incidence of Postpartum Hemorrhage in Pregnant Women with Placenta Previa: A Systematic Review and Meta-Analysis. PloS one 2017;12:e0170194.

99. Fan D, Wu S, Liu L, et al. Prevalence of antepartum hemorrhage in women with placenta previa: a systematic review and meta-analysis. Scientific reports 2017;7:40320.

100. Fan D, Li S, Wu S, et al. Prevalence of abnormally invasive placenta among deliveries in mainland China: A PRISMA-compliant Systematic Review and Meta-analysis. Medicine 2017;96:e6636.

101. van den Akker T, Brobbel C, Dekkers OM, Bloemenkamp KWM. Prevalence, Indications, Risk Indicators, and Outcomes of Emergency Peripartum Hysterectomy Worldwide: A Systematic Review and Meta-analysis. Obstetrics and gynecology 2016;128:1281-94.

102. Rodger MA, Gris J-C, de Vries JIP, et al. Low-molecular-weight heparin and recurrent placenta-mediated pregnancy complications: a meta-analysis of individual patient data from randomised controlled trials. The Lancet 2016;388:2629-41.

103. Qin JB, Wang H, Sheng X, Xie Q, Gao S. Assisted reproductive technology and risk of adverse obstetric outcomes in dichorionic twin pregnancies: a systematic review and meta-analysis. Fertility and sterility 2016;105:1180-92.

104. Qin J, Liu X, Sheng X, Wang H, Gao S. Assisted reproductive technology and the risk of pregnancy-related complications and adverse pregnancy outcomes in singleton pregnancies: a meta-analysis of cohort studies. Fertility and sterility 2016;105:73-85 e1-6.

105. Leone Roberti Maggiore U, Ferrero S, Mangili G, et al. A systematic review on endometriosis during pregnancy: diagnosis, misdiagnosis, complications and outcomes. Human reproduction update 2016;22:70-103.

106. Fan D, Wu S, Wang W, et al. Prevalence of placenta previa among deliveries in Mainland China: A PRISMA-compliant systematic review and meta-analysis. Medicine 2016;95:e5107.

107. Di Mascio D, Magro-Malosso ER, Saccone G, Marhefka GD, Berghella V. Exercise during pregnancy in normal-weight women and risk of preterm birth: a systematic review and meta-analysis of randomized controlled trials. American journal of obstetrics and gynecology 2016;215:561-71.

108. Qin J, Wang H, Sheng X, Liang D, Tan H, Xia J. Pregnancy-related complications and adverse pregnancy outcomes in multiple pregnancies resulting from assisted reproductive technology: a meta-analysis of cohort studies. Fertility and sterility 2015;103:1492-508 e1-7.

109. Marchi J, Berg M, Dencker A, Olander EK, Begley C. Risks associated with obesity in pregnancy, for the mother and baby: a systematic review of reviews. Obesity reviews : an official journal of the International Association for the Study of Obesity 2015;16:621-38.

110. Duffy JMN, Mylan S, Showell M, Wilson MJA, Khan KS. Pharmacologic intervention for retained placenta: a systematic review and meta-analysis. Obstetrics and gynecology 2015;125:711-18.

111. Chibueze EC, Parsons AJ, Ota E, Swa T, Oladapo OT, Mori R. Prophylactic antibiotics for manual removal of retained placenta during vaginal birth: a systematic review of observational studies and meta-analysis. BMC pregnancy and childbirth 2015;15:313.

112. Tersigni C, Castellani R, de Waure C, et al. Celiac disease and reproductive disorders:

meta-analysis of epidemiologic associations and potential pathogenic mechanisms. Human reproduction update 2014;20:582-93.

113. KLAR M, MICHELS KB. Cesarean section and placental disorders in subsequent pregnancies--a meta-analysis. Journal of perinatal medicine 2014;42:571-83.

114. GREENWOOD DC, THATCHER NJ, YE J, et al. Caffeine intake during pregnancy and adverse birth outcomes: a systematic review and dose-response meta-analysis. European journal of epidemiology 2014;29:725-34.

115. MENG X, XIE L, SONG W. Comparing the diagnostic value of ultrasound and magnetic resonance imaging for placenta accreta: a systematic review and meta-analysis. Ultrasound in medicine & biology 2013;39:1958-65.

116. MAHESHWARI A, KALAMPOKAS T, DAVIDSON J, BHATTACHARYA S. Obstetric and perinatal outcomes in singleton pregnancies resulting from the transfer of blastocyst-stage versus cleavage-stage embryos generated through in vitro fertilization treatment: a systematic review and meta-analysis. Fertility and sterility 2013;100:1615-21 e1-10.

117. CRESSWELL JA, RONSMANS C, CALVERT C, FILIPPI V. Prevalence of placenta praevia by world region: a systematic review and meta-analysis. Tropical medicine & international health : TM & IH 2013;18:712-24.

118. RAO KP, BELOGOLOVKIN V, YANKOWITZ J, SPINNATO JA, 2ND. Abnormal placentation: evidence-based diagnosis and management of placenta previa, placenta accreta, and vasa previa. Obstetrical & gynecological survey 2012;67:503-19.

119. FEKETE K, BERTI C, TROVATO M, et al. Effect of folate intake on health outcomes in pregnancy: a systematic review and meta-analysis on birth weight, placental weight and length of gestation. Nutrition journal 2012;11:75.

120. ABOU-NASSAR K, CARRIER M, RAMSAY T, RODGER MA. The association between antiphospholipid antibodies and placenta mediated complications: a systematic review and meta-analysis. Thrombosis research 2011;128:77-85.

121. RODGER MA, BETANCOURT MT, CLARK P, et al. The association of factor V leiden and prothrombin gene mutation and placenta-mediated pregnancy complications: a systematic review and meta-analysis of prospective cohort studies. PLoS medicine 2010;7:e1000292.

122. ZDOUKOPOULOS N, ZINTZARAS E. Genetic risk factors for placental abruption: a HuGE review and meta-analysis. Epidemiology 2008;19:309-23.

123. CONDE-AGUDELO A, ROSAS-BERMUDEZ A, KAFURY-GOETA AC. Effects of birth spacing on maternal health: a systematic review. American journal of obstetrics and gynecology 2007;196:297-308.

124. CASTLES A, ADAMS EK, MELVIN CL, KELSCH C, BOULTON ML. Effects of smoking during pregnancy. Five meta-analyses. American journal of preventive medicine 1999;16:208-15.

125. HULSE GK, MILNE E, ENGLISH DR, HOLMAN CD. Assessing the relationship between maternal cocaine use and abruptio placentae. Addiction 1997;92:1547-51.

126. ANANTH CV, SMULIAN JC, VINTZILEOS AM. The association of placenta previa with history of cesarean delivery and abortion: a metaanalysis. American journal of obstetrics and gynecology 1997;177:1071-8.

127. ANANTH CV, SAVITZ DA, WILLIAMS MA. Placental abruption and its association with hypertension and prolonged rupture of membranes: a methodologic review and meta-analysis. Obstetrics and gynecology 1996;88:309-18.

Supplementary file 3: AMSTAR-2 Results.

	AMSTAR-2																
Study	Q1	Q2	Q3	Q4	Q5	Q6	Q7	Q8	Q9	Q10	Q11	Q12	Q13	Q14	Q15	Q16	Quality
Sugai S, 2023	Y	Y	Y	Y	Y	Y	Y	Y	Y	Y	Y	Y	Y	Y	Y	Y	High
Busnelli A, 2022	Y	Y	Y	Y	Y	Y	Y	Y	Y	Y	Y	Y	Y	Y	Y	Y	High
Li L, 2022	Y	N	Y	Y	Y	Y	Y	Y	Y	Y	Y	Y	Y	Y	Y	Y	Low
Iqbal K, 2022	Y	N	Y	Y	Y	Y	Y	Y	Y	Y	Y	Y	Y	Y	Y	Y	Low
Jansen C, 2022	Y	Y	Y	Y	Y	Y	Y	Y	Y	Y	Y	Y	Y	Y	Y	Y	High
Jenabi E, 2022	Y	N	Y	Y	Y	Y	Y	Y	Y	Y	Y	Y	Y	Y	Y	Y	Low
Zhuo L, 2022	Y	N	Y	Y	Y	Y	Y	Y	Y	Y	Y	Y	Y	Y	Y	Y	Low
Steane SE, 2021	Y	Y	Y	Y	Y	Y	Y	Y	Y	Y	Y	Y	Y	Y	Y	Y	High
Matsuzaki S, 2021	Y	Y	Y	Y	Y	Y	Y	Y	Y	Y	Y	Y	Y	Y	Y	Y	High
Karacam Z, 2021	Y	Y	Y	Y	Y	Y	Y	Y	Y	Y	Y	Y	Y	Y	Y	Y	High
Matsuzaki S, 2021	Y	N	Y	Y	Y	Y	Y	Y	Y	Y	Y	Y	Y	Y	Y	Y	Low
Matsuzaki S, 2021	Y	N	Y	Y	Y	Y	Y	Y	Y	Y	Y	Y	Y	Y	Y	Y	Low
Iacovelli A, 2020	Y	Y	Y	Y	Y	Y	Y	Y	Y	Y	Y	Y	Y	Y	Y	Y	High
Hou YP, 2020	Y	Y	Y	Y	Y	Y	Y	Y	Y	Y	Y	Y	Y	Y	Y	Y	High
Tandon P, 2020	Y	N	Y	Y	Y	Y	Y	Y	Y	Y	Y	Y	Y	Y	Y	Y	Low
Horton J, 2019	Y	Y	Y	Y	Y	Y	Y	Y	Y	Y	Y	Y	Y	Y	Y	Y	High
Jenabi E, 2019	Y	N	Y	Y	Y	Y	Y	Y	Y	Y	Y	Y	Y	Y	Y	Y	Low
Balayla J, 2019	Y	N	Y	Y	Y	Y	Y	Y	Y	Y	Y	Y	Y	Y	Y	Y	Low
Martinelli KG, 2018	Y	Y	Y	Y	Y	Y	Y	Y	Y	Y	Y	Y	Y	Y	Y	Y	High
Sha T, 2018	Y	N	Y	Y	Y	Y	Y	Y	Y	Y	Y	Y	Y	Y	Y	Y	Low
Karami M, 2018	Y	N	Y	Y	Y	Y	Y	Y	Y	Y	Y	Y	Y	Y	Y	Y	Low
Roque M, 2018	Y	N	Y	Y	Y	Y	Y	Y	Y	Y	Y	Y	Y	Y	Y	Y	Low
Shobeiri F, 2017	Y	N	Y	Y	Y	Y	Y	Y	Y	Y	Y	Y	Y	Y	Y	Y	Low

Karami M, 2017	Y	N	Y	Y	Y	Y	Y	Y	Y	Y	Y	Y	Y	Y	Y	Y	Low
Ruiter L, 2016	Y	Y	Y	Y	Y	Y	Y	Y	Y	Y	Y	Y	Y	Y	Y	Y	High
Vahanian SA, 2015	Y	N	Y	Y	Y	Y	Y	Y	Y	Y	Y	Y	Y	Y	Y	Y	Low
Yin XA, 2015	Y	N	Y	PY	Y	Y	Y	Y	Y	Y	Y	Y	Y	Y	Y	Y	Critically low
Wang G, 2014	Y	N	Y	Y	Y	Y	Y	Y	Y	Y	Y	Y	Y	Y	Y	Y	Low
Huang QT, 2014	Y	N	Y	Y	Y	Y	Y	Y	Y	Y	Y	Y	Y	Y	Y	Y	Low
Grady R, 2012	Y	N	Y	Y	Y	Y	Y	Y	Y	Y	Y	Y	Y	Y	Y	Y	Low
Gurol-Urganci I, 2011	Y	N	Y	Y	Y	Y	Y	Y	Y	Y	Y	Y	Y	Y	Y	Y	Low
Saraswat L, 2010	Y	N	Y	Y	Y	Y	Y	Y	Y	Y	Y	Y	Y	Y	Y	Y	Low
Heslehurst N, 2008	Y	N	Y	Y	Y	Y	Y	Y	Y	Y	Y	Y	Y	Y	Y	Y	Low
Faiz AS, 2003	Y	N	Y	PY	Y	Y	Y	Y	Y	Y	Y	Y	Y	Y	Y	Y	Critically low

Q1: Did the research questions and inclusion criteria for the review include the components of PICO?

Q2: Did the report of the review contain an explicit statement that the review methods were established prior to the conduct of the review and did the report justify any significant deviations from the protocol?

Q3: Did the review authors explain their selection of the study designs for inclusion in the review?

Q4: Did the review authors use a comprehensive literature search strategy?

Q5: Did the review authors perform study selection in duplicate?

Q6: Did the review authors perform data extraction in duplicate?

Q7: Did the review authors provide a list of excluded studies and justify the exclusions?

Q8: Did the review authors describe the included studies in adequate detail?

Q9: Did the review authors use a satisfactory technique for assessing the risk of bias (RoB) in individual studies that were included in the review?

Q10: Did the review authors report on the sources of funding for the studies included in the review?

Q11: If meta-analysis was performed did the review authors use appropriate methods for statistical combination of results?

Q12: If meta-analysis was performed, did the review authors assess the potential impact of RoB in individual studies on the results of the meta-analysis or other evidence synthesis?

Q13: Did the review authors account for RoB in individual studies when interpreting/discussing the results of the review?

Q14: Did the review authors provide a satisfactory explanation for, and discussion of, any heterogeneity observed in the results of the review?

Q15: If they performed quantitative synthesis did the review authors carry out an adequate investigation of publication bias (small study bias) and discuss its likely impact on the results of the review?

Q16: Did the review authors report any potential sources of conflict of interest, including any funding they received for conducting the review?

Supplementary file 4. The detailed information of the factors and outcomes for placental anomalies (PP, PAS, and VP)

Placenta previa factors

AMA (> 45 years)

The authors to identify trends in pregnancy outcomes among pregnant patients older than 45 years. Fourteen included sutudies, involving 101794 placenta previa and 14604565 participants, found pregnant patients aged > 45 years had a significantly higher placenta previa (OR 3.61, 95% 2.70-4.81)[1].

ART (Programmed Frozen ET)

Ten studies were included and found a higher risk of PP in programmed frozen embryo transfer (PC-FET) (OR, 1.27, 95% CI 1.05-1.54, p = 0.01, fixed effects model) (very low quallity)[2]. After adjusted for maternal age, six studies were included and found the absence of an association (OR 1.16, 95% CI, 0.89-1.50, p = 0.27, fixed effects model). Four studies reported OR adjusted for maternal BMI (OR 1.28, 95% CI, 0.90-1.80, p = 0.17, fixed effects model). Two studies reported OR adjusted for the fertilization method (OR 0.84, 95% CI, 0.49-1.43, p = 0.52, fixed effects model). Three studies reported OR adjusted for embryo culture duration (OR 0.93, 95% CI, 0.66-1.31, p = 0.68, fixed effects model). Five studies reported OR adjusted for embryo biopsy (OR 1.58, 95% CI, 1.21-2.07, p = 0.0009, fixed effects model). Four studies reported OR adjusted for the indicationi to IVF/ICS (OR 1.06, 95% CI, 0.78-1.46, p = 0.70, fixed effects model).

IMH

IMH was not association with placenta previa in their meta-analysis[3]. They found the OR was 1.79, however, the 95%CI was 0.64 to 5.01 in their study, which including four studies.

Maternal alcohol

There is no association between prenatal alcohol consumption and placenta previa (aOR, 1.14, 95%CI, 0.84-1.56, p = 0.39) in the meta-analysis which was conducted on the four studies[4]. The true OR for similar future would be expected to lie within the 95% prediction interval, which was calculated to be 0.48-2.68.

Endometriosis

Overall, 19 studies examined the effect of endometriosis on the incidence of placenta previa in the meta-analysis[5]. They found endometriosis was associated with an increased incidence of placenta previa. The OR was 3.65 (95% CI 3.09-4.31) in the unadjusted pooled analysis (n=19) and 3.17 (95% CI 2.58-3.89) in the adjusted pooled analysis (n=12). In the sensitivity analysis in histologically confirmed endometiriosis, they found histologically confirmed endometriosis was correlated with a higher rate of placenta previa in the unadjusted pooled analysis (OR 4.99, 95% CI, 3.30-7.53; n=10) and in the adjusted pooled analysis (OR 4.23, 95% CI, 1.74-10.30; n=4).

Adolescent pregnancy

Seven of the studies reviewed reported results on the placenta previa in their meta-analysis[6]. And, the authors found placenta previa was less common in adolescent pregnancies (OR 0.52, 95% CI 0.31-0.88).

Prior UAE

According to the unadjusted pooled analysis, women who underwent prior UAE had higher rates of placenta previa (OR 5.62, 95% CI 1.48-21.34; n=3) than women who did not undgergo UAE[7]. However, according to the patient background-matched pooled analysis, women who underwent prior UAE did not have higher rates of placenta previa (OR 2.31, 95% CI 0.35-15.22) than women who did

not undergo prior UAE.

Anti-TNF in IBD

In patients with IBD, the authors found compared to patients not anti-TNF naïve during pregnancy, the pooled OR of placenta previa in those exposed to anti-TNF therapy was 1.58 (95% CI 0.30-8.47)[8].

Uterine leiomyoma

There was significant association between the uterine leiomyoma and the risk of placenta previa in studies adjusted (OR 2.21, 95% CI 1.48-2.94) in the meta-analysis[9]. However, this association in crude studies not significant (OR 0.98, 95% CI 0.06-1.90). Also, the results of subgroup analysis showed that uterine leiomyoma 5cm or more increased the risk of placenta previa by 3.53 fold compared with leiomyoma less than 5cm (OR 3.53, 95% CI 1.02-6.05).

ART (endometriosis)

The authors found women with endometriosis who did ART had higher rates of placenta previa (OR 3.31, 95% CI 1.26-8.71) than women with endometriosis who did not ART in their meta-analysis[10].

AMA (> 35 years)

The authors investigated the existence and magnitude of the association between advanced maternal age and occurrence of placenta previa in their meta-analysis[11]. They found an increase in age increased the magnitude of association strength (OR 3.16, 95% 2.79-3.57). In the analysis of subgroups by age group, as age increases, the chance of occurrence of PP also increase, OR = 2.70 (95% CI 2.30-3.10) in studies that used the 35-39 age range; for the >= 40 age range, the chance was 3.80 (95% CI 3.00-4.80).

ART (S) and ART (T)

The authors estimated the associated between placenta previa and ART in singleton and twin pregnancies in their meta-analysis[12]. According to their results, there was a significant association between ART and placenta previa. The overall estimate of OR was 2.67 (95% CI 2.01-3.34) and RR was 3.62 (95% CI 0.21-7.03) based on singleton pregnancies. The overall estimate of OR was 1.50 (95% CI 1.26-1.74) based on twin pregnancies. Based on studies estimating OR in singleton in crude studies, 2.78 (95% CI 1.89-3.67) and in adjusted studies was 2.59 (95% CI 1.70-3.48). Based on studies estimating OR in twins in crude studies, 1.47 (95% CI 1.23-1.71) and in adjusted studies was 2.91 (95% CI 1.08-4.73). There was no significant difference between adjusted and crude studies. However, there was significant difference between the results of high- and low-quality studies in singleton pregnancies.

ART (fresh ET)

There were six matched cohort studies with a high-quality score on the NOS scales in their meta-analysis[13]. They found a lower risk of placenta previa (RR 0.61, 95% CI 0.43-0.88) in pregnancies after frozen embryo transfer than after fresh embryo transfer.

Maternal smoking

Fourteen cohort studies and 7 case-control studies with 9094443 participants were included in their meta-analysis[14]. Based on the random effect model, compared to nonsmoker women, the estimated OR and RR of placenta previa was 1.42 (95% CI 1.30-1.54) and 1.27 (95% CI 1.18-1.35), respectively.

Prior abortion (S) and Prior abortion (I)

The authors found 3 cohort studies and 17 case-control studies with 2134529 participants in their meta-analysis[15]. They reported that there was a significant association between prior spontaneous abortion and the risk of placenta previa (OR 1.77, 95% CI 1.60-1.94) and between prior induced abortion and the risk of placenta previa (OR 1.36, 95% CI 1.02-1.69).

HDP

Seven cohort studies were included in their meta-analysis[16]. When all study results were pooled into the meta-analysis, a significantly inverse correlation between placenta previa and HDP was found (RR 0.55, 95% CI 0.32-0.97). For subgroup analyses, the same results were found in pregnancy-induced hypertension group (RR 0.36, 95% CI 0.23-0.57) but not in other HDPs group (RR 0.94, 95% CI 0.44-2.00). Sensitive analysis showed that all results were not materially altered.

Maternal asthma

The authors investigated if maternal asthma is associated with an increased risk of maternal and placental complications in preganncy in their meta-analysis[17]. Women with asthma had a significantly increased risk of placenta previa compared to women without asthma (RR 1.23, 95% CI 1.07-1.40; n=8). This increased risk remained significant after adjustment for covariates in five studies (OR 1.39, 95% CI 1.17-1.67).

CHB infection

Five studies involving 9088 placenta previa cases were identified in their meta-analysis[18]. No significant association between chronic hepatitis B virus infection and placenta previa was identified (OR 0.98, 95% CI 0.60-1.62).

eSET

Compared with spontaneously conceived singletons, elective single embryo transfer gestations had higher risks of placenta previa (RR 6.02, 95% CI 2.79-13.01; n=1) in their meta-analysis[19].

Prior CS

In the meta-analysis of 37 previously published studies from 21 countries, the authors found the overall pooled random effects odds ratio was 2.20 (95% CI 1.96-2.46)[20]. After adjustment, CS at first birth remained associated with an increased risk of placenta previa (OR 1.60, 95% CI 1.44-1.76). For the six population-based cohort studies, the results was 1.51 (95% CI 1.39-1.65).

Miscarriage (T)

In the meta-analysis, the authors found women with threatened miscarriage were more likely to have placenta previa (OR 1.62, 95% CI 1.19-2.22)[21].

Maternal obesity

In their study, the authors aimed to investigate relationships between obesity and impact on obstetric care[22]. The meta-analysis showed significant slightly reduced odds for placenta previa in obese women (OR 0.83, 95% CI 0.71-0.96).

Maternal cocaine use, male fetus, and preeclampsia

The authors performed a systematic review of the literature to estimate the placenta previa and its risk factors[23]. Three case-control studies demonstrated a higher risk of placenta previa with cocaine use during pregnancy (OR 2.9, 95% CI 1.9-4.3). Male fetuses were associated with higher risk of placenta previa (OR 1.2, 95% CI 1.1-1.3) in seven studies. Preeclampsia showed a protective effect on the risk of placenta previa (OR 0.9; n=3). However, there was no significantly.

Placenta previa outcomes

Blood transfusfusion (CS)

The study conducted to identify risk factors associated with blood transfusion in women undergoing cesarean section. In their meta-analysis, the authors found that placenta previa was found to be significanlty higher (OR 9.54, 95% CI 7.23-12.59; n=17) in the transfused group as compared to the non-transfused group[24].

Preterm delivery

In the meta-analysis, the authors found women with placenta previa were more likely to have a preterm birth before 37 weeks of gestation (risk difference 0.37, 95% CI 0.31-0.42), and before 34, 32, and 28 weeks of gestation ((OR 6.12, 95% CI 4.29-8.72), (OR 8.58, 95% CI 6.35-11.58), and (OR 5.61, 95% CI 4.02-7.83), respectively) than women without placenta previa[25].

IUGR

In the meta-analysis of over 1593226 singleton pregnancies and 10575 confirmed cases of placenta previa in 13 studies, the authors found that pregnancies with placenta previa were associated with a mild increase in the risk of IUGR, with a pooled OR of 1.19 (95% CI 1.10-1.27)[26].

Other outcomes

In the meta-analysis, the authors sought to evaluate the extent of the association between placenta previa and adverse pregnancy outcomes in singleton gestations[27]. In the comparative studies, placenta previa was significantly associated with lower 1- (RR 3.14, 95% CI 1.69-5.85; n=2) and 5-minute (RR 2.73, 95% CI 2.25-3.29; n=3) Apgar scores, NICU admissions (RR 4.09, 95% CI 2.80-5.97; n=5), and neonatal (RR 5.44, 95% CI 3.03-9.78; n=3) and perinatal (RR 3.01, 95% CI 1.41-6.43; n=3) death. However, there ws no significantly between placenta previa and SGA (RR 1.01, 95% CI 0.62-1.65; n=5).

PAS factors

Maternal Smoking

Fourteen studies were included in a meta-analysis[28]. Based on the random effect model, the estimated OR of the risk of PAS associated with smoking was 1.21 (95% CI: 1.02, 1.41). Subgroup analysis was conducted based on study design, and the result showed that the association between smoking and PAS among cohort studies was significant 1.35 (95% CI: 1.15, 1.55), but this association among case-control studies was not significant 0.83 (95% CI: 0.47, 1.19). In addition, there was no significant association between smoking and PAS based on crude/adjust form.

HDP

A meta-analysis was performed to evaluate the potential effects of hypertension in pregnancy on the placenta accreta spectrum[29]. The authors found pregnancy-induced hypertension was significantly related to lower prevalence of placenta accreta spectrum (OR 0.56, 95% CI 0.37-0.84; n=6) with moderate heterogeneity compared to pregnant women with no hypertension. When they looked at the result comparing the prevalence of hypertension in pregnancy in women with placenta accreta compared to no placenta accrete, they found that the placenta accreta spectrum was significantly related to lower prevalence of hypertension in pregnancy (OR 0.65, 95% CI 0.43-0.98) with moderate heterogeneity compared to no placenta accrete.

Prior UAE

Three comparator studies that compared the rate of PAS between women who did and did not undergo prior UAE. The unadjusted pooled analysis demonstrated that women with prior UAE had a higher rate of PAS (OR 28.47, 95% CI 7.61-106.57; n=3) than those who did not undergo prior UAE[7]. In the adjusted pooled analysis (all women had PPH during their previous delivery), prior UAE was still associated with PAS (OR 20.82, 95% CI 3.27-132.41; n=2).

ART

The meta-analysis aimed to explore the relation between ART pregnancy and PAS[30]. The authors found the risk of PAS was significantly higher in women who conceived with ART than in those with spontaneous conception (OR 5.03, 95% CI 3.34-7.56; n=9). In the sensitivity analysis accounting for

the type of embryo transfer, frozen embryo transfer was associated with an increased risk of PAS (OR 4.60, 95% CI 3.42-6.18; n=3) compared to fresh embryo transfer.

Maternal alcohol

The authors only found a single study reported on the prenatal alcohol consumption and PAS in their meta-analysis[4]. And, they indicated that there is no association between prenatal alcohol consumption and PAS (aOR 2.61, 95% CI 0.81-5.44). But, a robust increase in the likelihood of this complication when alcohol was considered as a dichotomous predictor.

ART (frozen-thawed ET)

In the meta-analysis, the authors found that the risk of placenta accreta development increased significantly in the frozen-thawed embryo transfer group compared to the fresh embryo transfer group (aOR 3.51, 95% CI 2.04-6.05; n=2)[31].

Multiple gestations, male fetus, and low SES

The authors described the association between potential influencing factors and PAS in their meta-analysis[32]. A synthesis of the results showed the OR with 95% CI of influencing factors were as follows: multiple gestations 1.90 (1.26-2.88) (n=7), male fetus 0.79 (0.74-0.84) (n=5), and low SES 0.51 (0.37-0.71) (n=3). Sensitivity analysis results suggested that the results of the meta-analysis were robust.

Other factors

In the meta-analysis, the authors explored the strength of association between different maternal and pregnancy characteristics and the occurrence of PAS[33]. They found maternal obesity (OR 1.37, 95% CI 1.04-1.81; n=5), advanced maternal age (OR 3.13, 95% CI 1.40-6.97; n=17), multiparity (OR 2.49, 95% CI 1.71-3.62; n=19), placenta previa (OR 11.00, 95% CI 4.71-25.80; n=24), placenta previa and prior cesarean section (OR 12.00, 95% CI 1.64-88.00; n=12), prior cesarean section (OR 4.66, 95% CI 3.02-7.18; n=33), and prior uterine surgery (OR 4.42, 95% CI 2.96-6.59; n=34) were associated with a higher risk of PAS. However, maternal smoking (OR 1.13, 95% CI 0.88-1.47; n=11), prior elective cesarean section (OR 3.73, 95% CI 0.50-27.71; n=3), prior emergency cesarean section (OR 1.17, 95% CI 0.21-6.65; n=3), prior myomectomy (OR 0.76, 95% CI 0.35-1.66; n=9), prior abortion (OR 1.36, 95% CI 0.84-2.20; n=6), prior curettage (OR 1.87, 95% CI 0.96-3.64; n=16), and short interval between prior cesarean section and subsequent pregnancy (<23 months) (OR 1.81, 95% CI 0.72-4.58; n=2) were not associated with a higher risk of PAS.

Vase previa factors

The authors aimed to review the literature on the risk indicators for vase previa in their study[34]. In the comparative studies, second-trimester placenta previa (OR 18.97, 95% CI 6.13-58.68; n=4), velamentous cord insertion (OR 672.44, 95% CI 112.10-4033.58; n=2), assisted reproductive technologies (OR 18.95, 95% CI 6.61-54.34; n=2), bilobed placenta (OR 71.50, 95% CI 14.64-349.25; n=2), and cord insertion in the lower third of the uterus at first-trimester ultrasound (OR 279.28, 95% CI 1.51-51547.34; n=2) had an increased risk of vase previa. However, there ws no significantly between multiple gestations and vase previa (OR 2.66, 95% CI 0.80-8.79; n=3).

Vase previa outcomes

Only one comparetive study pertaining to vasa previa was identified in the meta-analysis[27]. In the study, vasa previa was significantly associated with preterm delivery (RR 3.36, 95% CI 2.76-4.09), SGA (RR 4.02, 95% CI 2.64-6.12), perinatal death (RR 4.52, 95% CI 2.77-7.39), and 5-min Apgar score <7 (RR 2.18, 95% CI 1.36-3.50).

References

1. Sugai S, Haino K, Yoshihara K, Nishijima K. Pregnancy outcomes at maternal age over 45 years: a systematic review and meta-analysis. American journal of obstetrics & gynecology MFM 2023:100885.
2. Busnelli A, Schirripa I, Fedele F, Bulfoni A, Levi-Setti PE. Obstetric and perinatal outcomes following programmed compared to natural frozen-thawed embryo transfer cycles: a systematic review and meta-analysis. Human reproduction 2022;37:1619-41.
3. Zhuo L, Wang Z, Yang Y, Liu Z, Wang S, Song Y. Obstetric and offspring outcomes in isolated maternal hypothyroxinaemia: a systematic review and meta-analysis. Journal of endocrinological investigation 2022.
4. Steane SE, Young SL, Clifton VL, Gallo LA, Akison LK, Moritz KM. Prenatal alcohol consumption and placental outcomes: a systematic review and meta-analysis of clinical studies. American journal of obstetrics and gynecology 2021;225:607 e1-07 e22.
5. Matsuzaki S, Nagase Y, Ueda Y, et al. The association of endometriosis with placenta previa and postpartum hemorrhage: a systematic review and meta-analysis. American journal of obstetrics & gynecology MFM 2021;3:100417.
6. Karacam Z, Kizilca Cakaloz D, Demir R. The impact of adolescent pregnancy on maternal and infant health in Turkey: Systematic review and meta-analysis. Journal of gynecology obstetrics and human reproduction 2021;50:102093.
7. Matsuzaki S, Lee M, Nagase Y, et al. A systematic review and meta-analysis of obstetric and maternal outcomes after prior uterine artery embolization. Scientific reports 2021;11:16914.
8. Tandon P, Govardhanam V, Leung K, Maxwell C, Huang V. Systematic review with meta-analysis: risk of adverse pregnancy-related outcomes in inflammatory bowel disease. Alimentary Pharmacology & Therapeutics 2020;51:320-33.
9. Jenabi E, Fereidooni B. The uterine leiomyoma and placenta previa: a meta-analysis. The journal of maternal-fetal & neonatal medicine : the official journal of the European Association of Perinatal Medicine, the Federation of Asia and Oceania Perinatal Societies, the International Society of Perinatal Obstet 2019;32:1200-04.
10. Horton J, Sterrenburg M, Lane S, Maheshwari A, Li TC, Cheong Y. Reproductive, obstetric, and perinatal outcomes of women with adenomyosis and endometriosis: a systematic review and meta-analysis. Human reproduction update 2019;25:592-632.
11. Martinelli KG, Garcia EM, Santos Neto ETD, Gama S. Advanced maternal age and its association with placenta praevia and placental abruption: a meta-analysis. Cadernos de saude publica 2018;34:e00206116.
12. Karami M, Jenabi E, Fereidooni B. The association of placenta previa and assisted reproductive techniques: a meta-analysis. The journal of maternal-fetal & neonatal medicine : the official journal of the European Association of Perinatal Medicine, the Federation of Asia and Oceania Perinatal Societies, the International Society of Perinatal Obstet 2018;31:1940-47.
13. Sha T, Yin X, Cheng W, Massey IY. Pregnancy-related complications and perinatal outcomes resulting from transfer of cryopreserved versus fresh embryos in vitro fertilization: a meta-analysis. Fertility and sterility 2018;109:330-42 e9.

14. Shobeiri F, Jenabi E. Smoking and placenta previa: a meta-analysis. The journal of maternal-fetal & neonatal medicine : the official journal of the European Association of Perinatal Medicine, the Federation of Asia and Oceania Perinatal Societies, the International Society of Perinatal Obstet 2017;30:2985-90.
15. Karami M, Jenabi E. Placenta previa after prior abortion: a meta-analysis. Biomedical Research and Therapy 2017;4:1441.
16. Yin XA, Liu YS. Association between placenta previa and risk of hypertensive disorders of pregnancy: a meta-analysis based on 7 cohort studies. European review for medical and pharmacological sciences 2015;19:2146-52.
17. Wang G, Murphy VE, Namazy J, et al. The risk of maternal and placental complications in pregnant women with asthma: a systematic review and meta-analysis. The journal of maternal-fetal & neonatal medicine : the official journal of the European Association of Perinatal Medicine, the Federation of Asia and Oceania Perinatal Societies, the International Society of Perinatal Obstet 2014;27:934-42.
18. Huang QT, Chen JH, Zhong M, et al. The risk of placental abruption and placenta previa in pregnant women with chronic hepatitis B viral infection: a systematic review and meta-analysis. Placenta 2014;35:539-45.
19. Grady R, Alavi N, Vale R, Khandwala M, McDonald SD. Elective single embryo transfer and perinatal outcomes: a systematic review and meta-analysis. Fertility and sterility 2012;97:324-31.
20. Gurol-Urganci I, Cromwell DA, Edozien LC, et al. Risk of placenta previa in second birth after first birth cesarean section: a population-based study and meta-analysis. BMC pregnancy and childbirth 2011;11:95.
21. Saraswat L, Bhattacharya S, Maheshwari A, Bhattacharya S. Maternal and perinatal outcome in women with threatened miscarriage in the first trimester: a systematic review. BJOG : an international journal of obstetrics and gynaecology 2010;117:245-57.
22. Heslehurst N, Simpson H, Ells LJ, et al. The impact of maternal BMI status on pregnancy outcomes with immediate short-term obstetric resource implications: a meta-analysis. Obesity reviews : an official journal of the International Association for the Study of Obesity 2008;9:635-83.
23. Faiz AS, Ananth CV. Etiology and risk factors for placenta previa: an overview and meta-analysis of observational studies. The journal of maternal-fetal & neonatal medicine : the official journal of the European Association of Perinatal Medicine, the Federation of Asia and Oceania Perinatal Societies, the International Society of Perinatal Obstet 2003;13:175-90.
24. Iqbal K, Iqbal A, Rathore SS, et al. Risk factors for blood transfusion in Cesarean section: A systematic review and meta-analysis. Transfusion clinique et biologique : journal de la Societe francaise de transfusion sanguine 2022;29:3-10.
25. Jansen C, van Dijk CE, Kleinrouweler CE, et al. Risk of preterm birth for placenta previa or low-lying placenta and possible preventive interventions: A systematic review and meta-analysis. Frontiers in endocrinology 2022;13:921220.
26. Balayla J, Desilets J, Shrem G. Placenta previa and the risk of intrauterine growth restriction (IUGR): a systematic review and meta-analysis. Journal of perinatal medicine 2019;47:577-84.
27. Vahanian SA, Lavery JA, Ananth CV, Vintzileos A. Placental implantation abnormalities

and risk of preterm delivery: a systematic review and metaanalysis. American journal of obstetrics and gynecology 2015;213:S78-90.

28. Jenabi E, Salehi AM, Masoumi SZ, Maleki A. Maternal Smoking and the Risk of Placenta Accreta Spectrum: A Systematic Review and Meta-Analysis. BioMed research international 2022;2022:2399888.

29. Li L, Liu L, Xu Y. Hypertension in pregnancy as a risk factor for placenta accreta spectrum: a systematic review incorporating a network meta-analysis. Archives of gynecology and obstetrics 2022.

30. Matsuzaki S, Nagase Y, Takiuchi T, et al. Antenatal diagnosis of placenta accreta spectrum after in vitro fertilization-embryo transfer: a systematic review and meta-analysis. Scientific reports 2021;11:9205.

31. Roque M, Valle M, Sampaio M, Geber S. Obstetric outcomes after fresh versus frozen-thawed embryo transfers: A systematic review and meta-analysis. JBRA assisted reproduction 2018;22:253-60.

32. Hou YP, Lommel L, Wiley J, et al. Influencing factors for placenta accreta in recent 5 years: a systematic review and meta-analysis. The journal of maternal-fetal & neonatal medicine : the official journal of the European Association of Perinatal Medicine, the Federation of Asia and Oceania Perinatal Societies, the International Society of Perinatal Obstet 2020:1-8.

33. Iacovelli A, Liberati M, Khalil A, et al. Risk factors for abnormally invasive placenta: a systematic review and meta-analysis. The journal of maternal-fetal & neonatal medicine : the official journal of the European Association of Perinatal Medicine, the Federation of Asia and Oceania Perinatal Societies, the International Society of Perinatal Obstet 2020;33:471-81.

34. Ruiter L, Kok N, Limpens J, et al. Incidence of and risk indicators for vasa praevia: a systematic review. BJOG : an international journal of obstetrics and gynaecology 2016;123:1278-87.
